# Efficacy of combined sodium-glucose cotransporter 2 inhibitors and finerenone in chronic kidney disease: a systematic review and meta-analysis

**DOI:** 10.3389/fphar.2026.1803971

**Published:** 2026-05-13

**Authors:** Chen-Fu Wen, Min-Hsiang Chuang, Vin-Cent Wu, Jui-Yi Chen

**Affiliations:** 1 Department of Internal Medicine, Chi Mei Medical Center, Tainan, Taiwan; 2 Division of Nephrology, Department of Internal Medicine, Chi Mei Medical Center, Tainan, Taiwan; 3 Division of Nephrology, Department of Internal Medicine, National Taiwan University Hospital, Taipei, Taiwan; 4 Department of Health and Nutrition, Chia Nan University of Pharmacy and Science, Tainan, Taiwan; 5 Department of Public Health, College of Medicine, National Cheng Kung University, Tainan, Taiwan

**Keywords:** cardiovascular outcome, chronic kidney disease, finerenone, hyperkalemia, meta-analysis, sodium-glucose cotransporter 2 inhibitor

## Abstract

**Background:**

Sodium-glucose cotransporter two inhibitors (SGLT2is) and finerenone have demonstrated individual efficacy in reducing cardiorenal events among patients with diabetic chronic kidney disease (CKD). However, the additive benefits and safety profile of combining these agents remain unclear.

**Methods:**

We conducted a systematic review and meta-analysis of randomized controlled trials and observational studies comparing finerenone plus SGLT2is *versus* monotherapy. Primary outcomes included all-cause mortality, major adverse cardiovascular events (MACEs), kidney-specific composite outcomes, and hyperkalemia risk. Pooled odds ratios (OR) and 95% confidence intervals (CIs) were calculated using a random-effects model.

**Results:**

A total of eight studies (N = 1,580) were included. Compared with finerenone monotherapy, combination therapy significantly reduced all-cause mortality (OR 0.58; 95% CI: 0.36–0.93). Furthermore, combination therapy also reduced MACE risk (OR 0.70; 95% CI: 0.51–0.97) and major adverse kidney event (MAKE) risk (OR 0.63; 95% CI: 0.44–0.89) compared with finerenone monotherapy. Combination therapy significantly reduced urinary albumin–creatinine ratio (UACR) more than finerenone monotherapy, with a mean difference of 0.10 (equivalent to a 10% greater reduction; combination vs. finerenone, 95% CI: 0.00–0.19; *p* = 0.045). However, the combined group had a higher risk of hyperkalemia compared to SGLT2i monotherapy (OR 3.00; 95%: CI 2.50–3.61). No significant benefit was observed in composite kidney outcomes compared with SGLT2 inhibitors alone.

**Conclusion:**

Combining finerenone with SGLT2i may improve survival and reduced risks of MACEs and MAKEs compared with finerenone monotherapy in patients with diabetic CKD. These findings support careful consideration of dual therapy, especially in high-risk populations.

**Systematic Review Registration:**

https://www.crd.york.ac.uk/prospero/display_record.php?ID=CRD420251023918, identifier: CRD420251023918.

## Introduction

Sodium-glucose cotransporter two inhibitors (SGLT2is) have demonstrated significant renal and cardiovascular protective effects in patients with CKD and type 2 diabetes mellitus (T2DM) (Heerspink et al., 2021). By reducing intraglomerular pressure, promoting natriuresis, and exerting anti-inflammatory effects, SGLT2is lower the risk of death, major adverse cardiovascular events (MACEs), and major adverse kidney events (MAKEs) (Kaze et al., 2022; Patel et al., 2024). These benefits extend beyond glycemic control and support the use of SGLT2is in the management of patients with CKD.

Mineralocorticoid receptor antagonists (MRAs), particularly finerenone, offer complementary renoprotective benefits through anti-inflammatory and anti-fibrotic mechanisms (Kolkhof et al., 2022). Unlike steroidal MRAs, finerenone provides a targeted reduction in albuminuria with a lower risk of hyperkalemia (Flack et al., 2023). However, while albuminuria reduction is a recognized surrogate marker of CKD progression, evidence on its impact on hard outcomes such as mortality, MACEs, and MAKEs remains limited.

Emerging studies suggest that combining SGLT2is with finerenone may provide additional benefits in patients with CKD (Agarwal et al., 2025). However, discrepancies exist in study designs and reported outcomes, making it unclear whether combination therapy significantly improves clinical endpoints. This meta-analysis aims to systematically evaluate the impact of combined SGLT2i and finerenone therapy on mortality, MACEs, MAKEs, and reduction in proteinuria, providing clarity on its potential role in optimizing treatment strategies for this high-risk population.

## Materials and methods

This meta-analysis was undertaken following the Preferred Reporting Items for Systematic Reviews and Meta-Analyses (PRISMA) 2020 guidelines (Page et al., 2021) and was registered in the PROSPERO database (registration number ID: CRD420251023918). The full PROSPERO protocol registration is provided in Supplementary Table S1. The full 2020 PRISMA checklist is provided in Supplementary Table S2.

### Search strategy

A systematic search of PubMed, EMBASE, and the Cochrane Library was performed from database inception to 6 June 2025, without limitations on language or geographic region. Further details are summarized in Supplementary Table S3.

### Inclusion and exclusion criteria

We included studies that met the following criteria: (1) adults (aged ≥18 years) with CKD, based on study-specific definitions of eGFR and proteinuria (Table 1); (2) intervention: combination therapy with finerenone and SGLT2i; (3) comparator: monotherapy with either finerenone or SGLT2i; and (4) outcomes: all-cause mortality, MACEs, MAKEs, reduction of urinary albumin–creatinine ratio (UACR), and hyperkalemia.

**TABLE 1:** Baseline characteristics of the included studies.

Study (year)	Study design	Age (yr)	Female (%)	Intervention group vs. control group	Patients Total/C/F/S (n)	CKD definition
FIGARO-DKD (2021)	RCT, double-blind, multiple centers	64.1 ± 9.8^£^	30.6^£^	Combined vs. finerenone or SGLT2i	T: 3,990 C: 314 F: 3,372 S: 304	1. UACR: 30–300 mg/g, and eGFR: 25–90 mL/min/1.73^2^ 2. UACR: 300–5000 mg/g, and eGFR: >60 mL/min/1.73^2^
FIDELIO-DKD (2022)	RCT, double-blind, multiple centers	65.6 ± 9.1^£^	29.8^£^	Combined vs. finerenone or SGLT2i	T: 2,968 C: 124 F: 2,709 S: 135	1. UACR: 30–300 mg/g, and eGFR: 25–60 mL/min/1.73^2^, with history of DM retinopathy 2. UACR: 300–5000 mg/g, and eGFR: 25–75 mL/min/1.73^2^
FIDELITY (2022)	Individual patient level data from the FIDELIO-DKD and FIGARO-DKD	64.8 ± 9.5	30.2	Combined vs. finerenone or SGLT2i	T: 6,958 C: 438 F: 6,081 S: 439	1. UACR: 30–5000 mg/g, and eGFR ≥ 25 mL/min/1.73^2^
Mårup et al. (2024)	Single-center, prospective randomized open-label clinical trial	60.0 ± 15.6	25	Combined vs. finerenone or dapagliflozin	T: 20 C^@^: 20 F: 20 D: 20	UACR: 150–2000 mg/g, and eGFR: 25–45 mL/min/1.73^2^
Chuang et al. (2025)	Retrospective cohort study	67.3 ± 11.3	39.4	Combine vs. finerenone or SGLT2i	T: 47,743 C: 853 F: 942 S: 45,948	1. eGFR: <60 mL/min/1.73^2^ 2. Proteinuria 3. UACR ≥30 mg/g 4. UPCR ≥150 mg/g
Hanouneh et al. (2024)	Retrospective cohort study	70.7 ± 6.0	37.8	Combine vs. finerenone or SGLT2i	T: 98 C: 24 F: 22 S: 52	UACR: ≥30 mg/g, and eGFR: 25–90 mL/min/1.73^2^
CONFIDENCE (2025)	RCT, double-blind	66.5 ± 10.3	24.8	Combined vs. finerenone or empagliflozin	T: 800 C^*^: 269 F: 264 E: 267	UACR: 100–5000 mg/g and eGFR: 30–90 mL/min/1.73^2^
Kovesdy et al. (2025)	Retrospective cohort study	70.3 ± 10.1	44.1^£^	Combine vs. finerenone	T: 15,948 C^*^: 1,402 F: 5,180	1. eGFR: 15–60 mL/min/1.73^2^ 2. UACR ≥30 mg/g

^*^ Combination of finerenone and empagliflozin

^@^ Combination of finerenone and dapagliflozin.

^£^ Refers to the total study population, while only a subset was included in this meta-analysis and the characteristics may not represent the analyzed group.

Abbreviations: C, combined group; D, dapagliflozin; DKD, diabetic kidney disease; E, empagliflozin; eGFR, estimated glomerular filtration; F, finerenone; RCT, randomized control trials; S, SGLT2i; SGLT2i, Sodium-glucose co-transporter two inhibitor; UACR, urinary albumin/creatinine ratio; UPCR, urinary protein/creatinine ratio.

Eligible study designs included RCTs and observational studies. Studies were excluded if they (1) were not RCTs or observational studies (e.g., protocols or abstracts); (2) did not assess all-cause mortality, MACEs, MAKEs, UACR, or hyperkalemia (e.g., focused only on atrial fibrillation risk (Filippatos et al., 2021)); or (3) enrolled only patients with dialysis-dependent kidney failure or kidney transplant recipients.

### Study selection and data extraction

Duplicated records were removed, and two reviewers (CFW and JYC) independently examined titles and abstracts, followed by full-text screening to determine study eligibility. We screened the titles and abstracts of all identified studies, followed by full-text assessment to determine eligibility. Data extraction was conducted independently by the same reviewers using a standardized, pilot-tested form. Discrepancies were resolved by consensus, with involvement of a third reviewer when necessary.

For the included RCTs, data were primarily extracted from pre-specified subgroup analyses within each study, focusing specifically on participants with CKD who received finerenone, SGLT2i, or their combination. The analysis included all types of SGLT2is, such as dapagliflozin, empagliflozin, and canagliflozin, as reported. Extracted data included detailed study characteristics (e.g., sex, age, study design, sample size, and definition of CKD), intervention arms (combination therapy), comparators (monotherapy of finerenone or SGLT2i), and outcomes of interest (e.g., all-cause mortality, composite kidney and cardiovascular outcomes, reduction of UACR, and hyperkalemia).

For studies in which one treatment arm reported zero events, a continuity correction of 0.5 was applied to both the event count and the total sample size in each group to allow for inclusion in the meta-analysis. When both arms reported zero events, the study was excluded from the pooled analysis as it provides no information on relative treatment effects (Higgins et al., 2023).

### Quality assessment

Methodological quality of the selected studies was evaluated using the Cochrane Risk of Bias Tool (ROB 2.0) (Sterne et al., 2019) for RCTs and Risk of Bias In Non-randomized Studies of Interventions (ROBINS-I) (Sterne et al., 2016) for observational studies. RCTs were judged as low risk, some concerns, or high risk of bias, while observational studies were judged as low, moderate, or serious risk of bias. Two reviewers (CFW and JYC) carried out the assessment independently. Any discrepancies were addressed through discussion.

### Outcomes

The primary outcome of this meta-analysis was all-cause mortality, and secondary outcomes were MACEs, MAKEs, and reduction in UACR. A MACE was defined as cardiovascular death, non-fatal myocardial infarction, non-fatal stroke, or hospitalization for heart failure. A MAKE was defined as ESKD requiring hemodialysis for >90 days, eGFR <15 mL/min/1.73 m^2^, kidney transplantation, a sustained ≥40% decline in eGFR for >4 weeks, or death from kidney disease. Studies that reported changes in UACR were also included to assess the effect on proteinuria reduction. In addition, the adverse event of hyperkalemia was also recorded (Supplementary Table S4). We rated the certainty of evidence according to Cochrane methods and the GRADE approach.

### Data synthesis and statistical analysis

Given the potential heterogeneity in population demographics, we adopted the random-effects model for outcome analysis. The results were presented as ORs with their associated 95% CIs. For continuous outcomes such as UACR reduction, we calculated the weighted mean difference (WMD) with 95% confidence intervals based on between-group differences in change from baseline. UACR changes were expressed on a proportion scale, and thus, the pooled WMD represents the absolute difference in percentage reduction between groups. When necessary, data were harmonized to a common scale. Statistical heterogeneity was evaluated using the chi-square test and the I^2^ statistic. Between-study variance (τ^2^) was estimated using the DerSimonian and Laird method, as implemented in the meta package in R. Given the considerable heterogeneity likely driven by observational studies among the eight included for hard outcomes, a sensitivity analysis was performed by excluding the observational studies to assess the consistency of the overall results. Funnel plots were generated to examine potential publication bias.

We applied a random-effects model to construct the cumulative Z curve. O’Brien-Fleming’s α-spending function was applied and converted into sequential monitoring boundaries, calculated with a significance level of 0.05 and a statistical power of 80% (Wetterslev et al., 2017). A relative risk reduction of 20% for mortality, MACE, and MAKE was used to define the futility boundaries. When the cumulative Z curve crossed the sequential boundaries or entered the futility zone, it was considered sufficient to support or reject the anticipated intervention effect, and no further analyses were conducted.

All statistical analyses were performed using the “meta” and “metafor” package in R software (Version 4.3.1, 21 April 2023). TSA software (version 0.9.5.5 b, reviewed November 2016) was used to evaluate the cumulative effects of randomized trials. A *p*-value <0.05 was deemed statistically significant.

## Results

### Study selection

**FIGURE 1:**
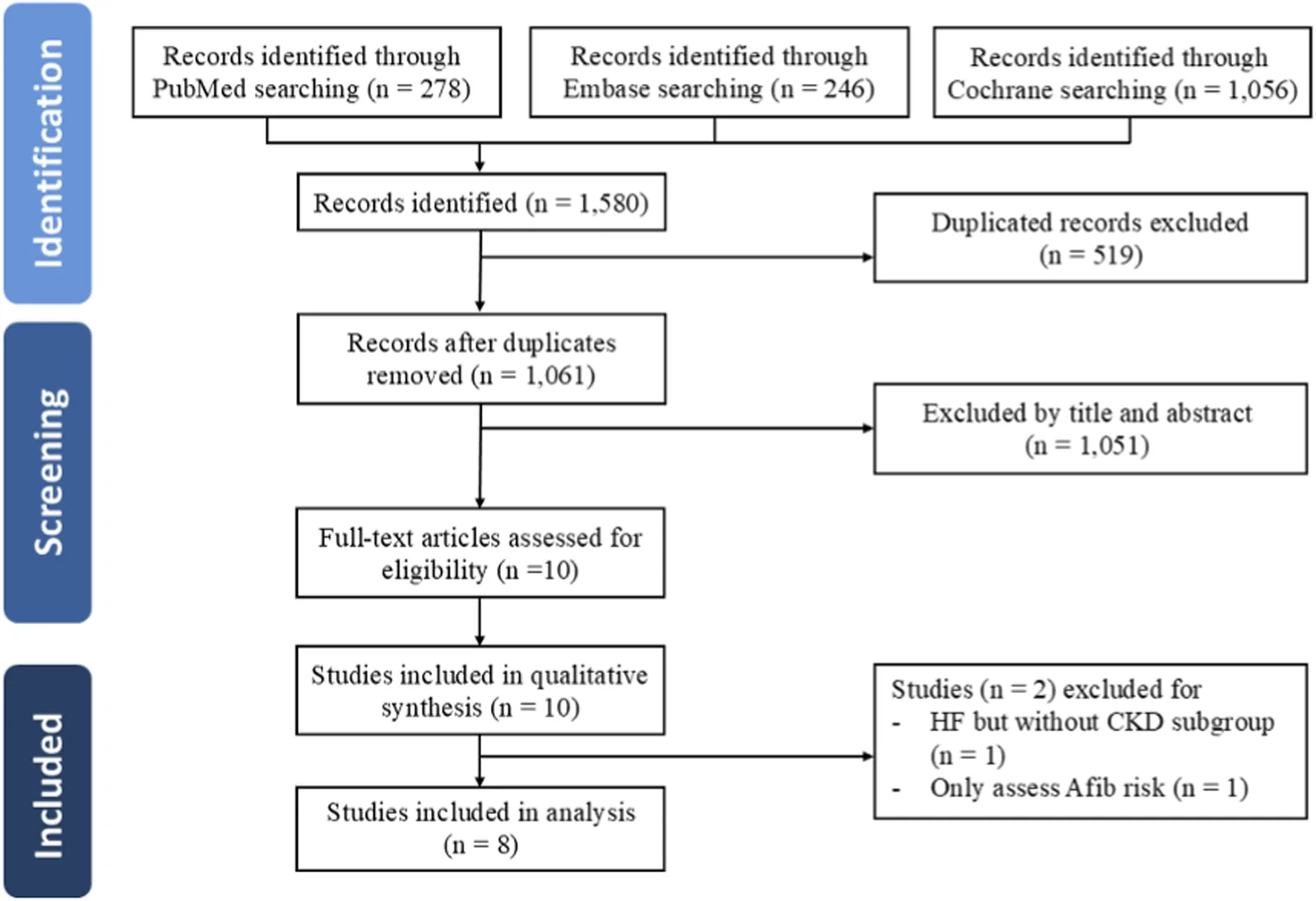
Preferred reporting items for systematic reviews and meta-analyses (PRISMA) flow diagram for the searching and identifying included studies. Abbreviations: Afib, atrial fibrillation; CKD, chronic kidney disease; HF, heart failure; PRISMA, preferred reporting items for systematic reviews and meta-analyses; RCT, randomized controlled trial.

An initial search identified 1,580 publications from electronic databases and manual searches. After removing duplicates (n = 519), 1,061 studies underwent title and abstract screening. The full text of 10 articles was retrieved for eligibility assessment. We excluded two articles for the following reasons: one study focused on heart failure (HF) without a CKD subgroup (Solomon et al., 2024), and one study assessed only atrial fibrillation (Afib) risk (Filippatos et al., 2021). Consequently, eight eligible studies were enrolled. A comprehensive PRISMA flow diagram is shown in Figure 1.

### Characteristics of the included studies

Baseline characteristics and outcomes of the included studies are summarized in Tables 1, 2. This meta-analysis included five RCTs and three retrospective cohort studies, encompassing a total of 71,567 participants. Since the FIDELITY study is a *post hoc* analysis of the FIGARO-DKD and FIDELIO-DKD trials, the participants should not be double-counted. The mean age of participants ranged from 60.0 to 70.7 years, and the proportion of female participants ranged from 24.8% to 44.1%. All studies enrolled individuals with CKD, although the definitions varied. Most studies defined CKD using thresholds of reduced eGFR (ranging from <60 to <90 mL/min/1.73 m^2^) and elevated albuminuria [urinary albumin/creatinine ratio (UACR); urinary protein/creatinine ratio (UPCR)], with some requiring additional criteria such as diabetic retinopathy or proteinuria diagnosis. Hyperkalemia was defined as a potassium level over 5 mmol per liter.

**TABLE 2:** Reported outcomes of the included studies.

Study (year)	Primary outcome	Secondary outcome	All-cause mortality n/total (%)	MAKE^#^ n/total (%)	UACR relative change (%) (95% CI)	MACE* n/total (%)	Hyperkalemia n/total (%)
FIGARO-DKD (2021)	Death from cardiovascular causes, non-fatal myocardial infarction, nonfatal stroke, hospitalization for HF	ESKD on HD > 90 days, eGFR <15 mL/min/1.73^2^, kidney transplantation, sustained eGFR decreased >40% from baseline for more than 4 weeks, or death from kidney disease	NR	C^&^: 6/314 (1.9) F: 102/3,372 (3.0) S: 11/304 (3.6)	NR	C^&^: 24/314 (7.6) F: 434/3,372 (12.9) S: 37/304 (12.2)	NR
FIDELIO-DKD (2022)	ESKD on HD > 90 days, eGFR <15 mL/min/1.73^2^, kidney transplantation, sustained eGFR decreased >40% from baseline for more than 4 weeks, or death from kidney disease	Death from cardiovascular causes, non-fatal myocardial infarction, nonfatal stroke, hospitalization for HF	NR	C^&^: 14/124 (11.3) F: 490/2,709 (18.1) S: 10/135 (7.4)	NR	C^&^: 15/124 (12.1) F: 352/2,709 (13.0) S: 15/135 (11.1)	NR
FIDELITY (2022)	CV composite outcomes: CV death, nonfatal myocardial infarction, nonfatal stroke, or hospitalization For heart failure Kidney composite outcomes: kidney failure, a sustained >57% decline in eGFR from baseline, or renal death	Hospitalization for heart failure All-cause death	C^&^: 20/438 (4.6) F: 532/6,081 (8.7) S: 30/439 (6.8)	NR	C: −0.63 (-0.57 to −0.70) F: −0.69 (-0.67 to −0.71)	NR	C^&^: 45/438 (10.3) F: 867/6,072 (14.3) S: 12/439 (2.7)
Chuang et al. (2025)	ESKD on HD > 90 days, eGFR <15 mL/min/1.73^2^, kidney transplantation, sustained eGFR decreased >40% from baseline for more than 4 weeks, or death from kidney disease	All-cause mortality Death from cardiovascular causes, non-fatal myocardial infarction, nonfatal stroke, hospitalization for HF	C^&^: 11/801 (1.4) F: 13/801 (1.6) C^&^: 16/1335 (1.2) S: 19/1335 (1.4)	C^&^: 15/643 (2.3) F: 12/643 (1.8) C^&^: 18/850 (2.1) S: 15/850 (1.7)	NR	C^&^: 16/801 (2.0) F: 22/801 (2.7) C^&^:25/1335 (1.9) S: 30/1335 (2.2)	C^&^: 299/2,298 (13.0) F: 147/2,298 (6.4) C^&^: 387/3,407 (11.4) S: 142/3,407 (4.2)
Hanouneh et al. (2024)	Percentage of patients achieving a >50% reduction in UACR from their baseline levels	The percentage of reduction in albuminuria at the end of the 8-month follow-up period Changes in serum potassium levels, and changes in eGFR	NR	NR	C: −73 (-53 to −92) F: −55 (-43 to −67) S: −45 (-32 to −58)	NR	NR
Mårup et al. (2024)	The change in UACR from randomization to 8 weeks among all participants measured as the average of two consecutive morning spot urine samples taken 1 day apart	Change in UACR, albuminuria measured on a 24 h urine sample	NR	NR	C: −36 (-46 to −24) F: −32 (-47 to −14) S: −39 (-52 to −22)	NR	NR
CONFIDENCE (2025)	Relative change in the log-transformed mean UACR from baseline to 180 days	The relative change in the UACR between the end-of-treatment visit and 30 days after the end-of-treatment visit AMI, cardiac death, death	C^&^: 0/268 (0) F: 0/264 (0) S: 1/266 (0.4%)	C^&^: 0/268 (0) F: 6/264 (2.3) E: 1/266 (0.4)	C: −52 (−46 to −56) F: −32 (−24 to −39) S: −29 (−21 to −36)	C^&^: 1/268 (0.4) F: 0/264 (0) E: 1/266 (0.4)	C^&^: 25/268 (9.3) F: 30/264 (11.4) E: 10/266 (3.8)
Kovesdy et al. (2025)	Changes from baseline in UACR, eGFR, and serum potassium were determined at 4- and 12-month follow up	An episode of hyperkalemia	NR	NR	C^&^: −47.4 (-85.6 to −9.2) F: −34.0 (-48.4 to −19.7)	NR	NR

^#^ MAKE: End stage kidney disease on hemodialysis more than 90 days, eGFR <15 mL/min/1.732, kidney transplantation, sustained eGFR, decreased more than 40% from baseline for more than 4 weeks, or death from kidney disease.

^*^ MACE: death from cardiovascular causes, non-fatal myocardial infarction, nonfatal stroke, hospitalization for heart failure.

^$^ Mean ± SD.

^&^ Combined group: finerenone and SGLT2i.

Abbreviations: AMI, acute myocardial infarction; C, combined; E, empagliflozin; eGFR, estimated glomerular filtration rate; ESKD, end-stage kidney disease; DKD, diabetic kidney disease; eGFR, estimated glomerular filtration rate; F, finerenone; HD, hemodialysis; NR, not reported; S, SGLT2i; SGLT2i, sodium-glucose co-transporter two inhibitor; UACR, urinary albumin–creatinine ratio; UPCR, urinary protein/creatinine ratio.

Five of the included RCTs administered finerenone at doses ranging from 10 to 20 mg once daily, while Chuang et al. (2025) did not report specific dosing information. Among the SGLT2i agents, the CONFIDENCE trial specified the use of empagliflozin, Marup et al. (2024) specified the use of dapagliflozin, and Hanouneh et al. (2024) mentioned the use of empagliflozin or dapagliflozin; the remaining studies allowed the use of various SGLT2i agents without restriction. All eight studies excluded patients with CKD stage 5 or end-stage kidney disease (ESKD). Additionally, four studies explicitly assessed MACE or MAKE as primary or secondary outcomes.

### Quality of enrolled studies

Three RCTs were rated as low risk of bias. The *post hoc* subgroup analysis (Filippatos et al., 2022) and one RCT (Marup et al., 2024) were assessed as having some concerns due to the absence of pre-specified protocol, particularly with respect to reporting bias. Chuang et al. (2025) was rated as having a moderate risk of bias because confounding control and medication adherence were identified as key potential sources of bias. Hanouneh et al. (2024) was rated as having a moderate risk of bias due to the absence of pre-specified protocol. Kovesdy et al. (2025) was rated as having a serious risk of bias as it was a descriptive report and lacked prior registration (Supplementary Figures S1A, S1B).

### Primary and secondary outcomes

**FIGURE 2:**
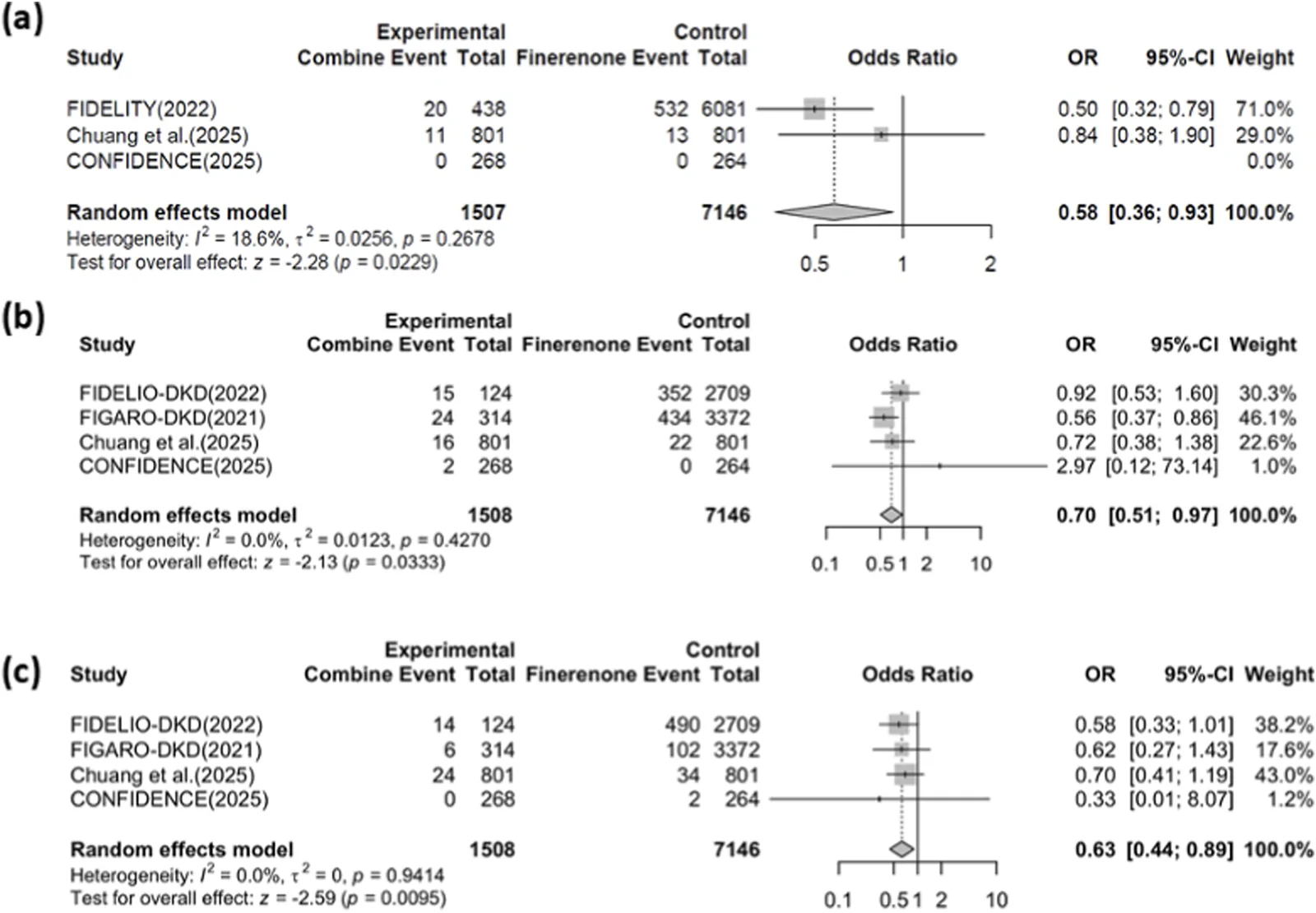
Forest plots comparing combined therapy *versus* finerenone monotherapy for **(a)** all-cause mortality, **(b)** MACE, and **(c)** MAKE. Abbreviations: CI, confidence interval; MACE, major adverse cardiovascular event; MAKE, major adverse kidney event; OR, odds ratio; SGLT2i, sodium-glucose co-transporter two inhibitor.

Compared with finerenone monotherapy, combination therapy significantly reduced the risk of all-cause mortality (combination vs. finerenone, OR = 0.58; 95% CI: 0.36–0.93; *p* = 0.02; I^2^ = 18.6%) (Figure 2a). However, no significant difference was observed when combination therapy was compared with SGLT2i monotherapy (combination vs. SGLT2i, OR = 0.73; 95% CI: 0.47–1.13; *p* = 0.16) (Supplementary Figure S2A). Funnel plots suggested possible publication bias (Supplementary Figures S3A, S4A).

In addition, combination therapy was associated with a lower risk of MACE compared to finerenone alone (combination vs. finerenone, OR = 0.70; 95% CI: 0.51–0.97; *p* = 0.03; I^2^ = 0%) (Figure 2b). Compared with SGLT2i monotherapy, the difference was not statistically significant (combination vs. SGLT2i, OR = 0.77; 95% CI: 0.55–1.08; *p* = 0.14) (Supplementary Figure S2B). Funnel plots again indicated potential publication bias (Supplementary Figures S3B, S4B).

Combination therapy significantly reduced the risk of MAKE relative to finerenone monotherapy (combination vs. finerenone, OR = 0.63; 95% CI: 0.44–0.89; *p* < 0.01; I^2^ = 0%) (Figure 2c). No significant difference was observed compared to SGLT2i monotherapy (combination vs. SGLT2i, OR = 0.87; 95% CI: 0.58–1.31; *p* = 0.51) (Supplementary Figure S2C). Evidence of publication bias was observed (Supplementary Figures S3C, S4C).

**FIGURE 3:**
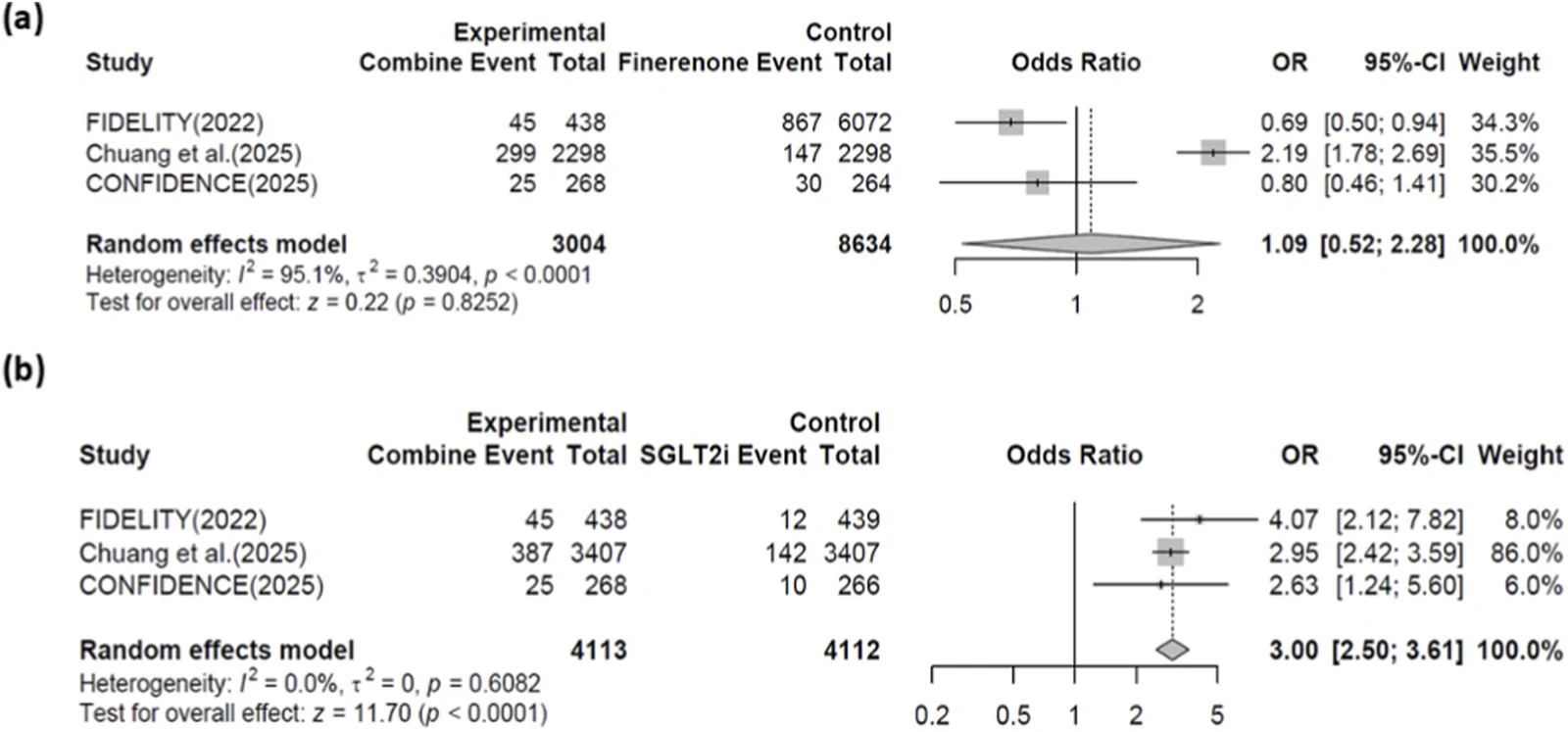
Forest plots comparing combined therapy versus **(a)** finerenone monotherapy and **(b)** SGLT2i monotherapy for UACR reduction. Abbreviations: CI, confidence interval; UACR, urine albumin-to-creatinine ratio; MD, mean difference; MMD, meta-analysis mean difference; SD, standard deviation; SGLT2i, sodium-glucose co-transporter two inhibitor.

In the overall analysis, the combined group had a mean UACR reduction of 54.3% ± 12.8%, compared with 44.4% ± 15.1% in the finerenone alone group. After pooling the results, combination therapy significantly reduced UACR more than finerenone monotherapy, with a mean difference of 0.10 (equivalent to a 10% greater reduction; combination vs. finerenone, 95% CI: 0.00–0.19; *p* = 0.045). However, the heterogeneity was extremely high (I^2^ = 99.9%), suggesting substantial inconsistency among the included studies (Figure 3). A sensitivity analysis was performed by excluding the observational studies to assess the consistency of the overall results, as illustrated in Supplementary Figures S5, S6.

**FIGURE 4:**
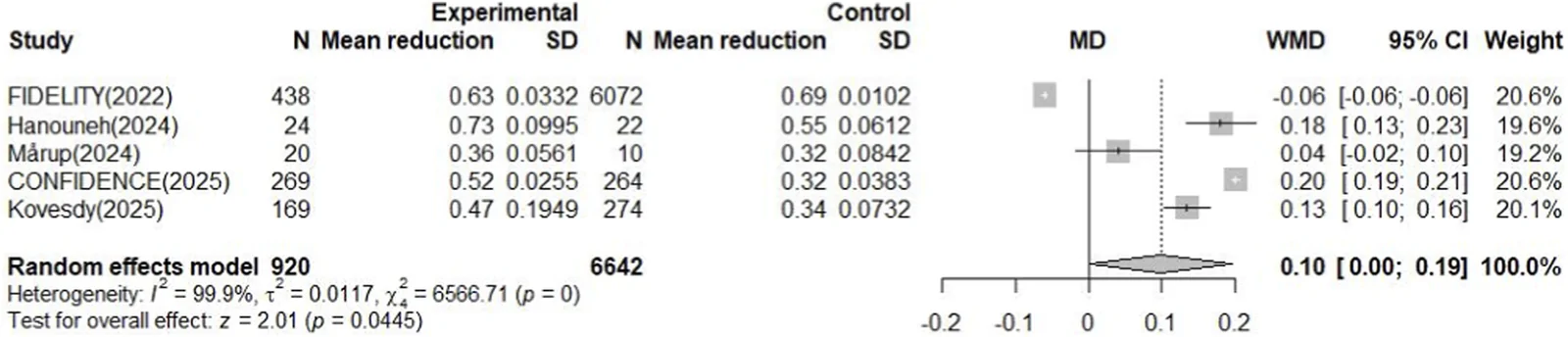
Forest plots comparing combined therapy *versus* SGLT2i monotherapy for risk of hyperkalemia. Abbreviations: CI, confidence interval; OR, odds ratio; SGLT2i, sodium-glucose co-transporter two inhibitor.

Compared to SGLT2i monotherapy, combination therapy was associated with a higher risk of hyperkalemia (combination vs. SGLT2i, OR = 3.00; 95% CI: 2.50–3.61; *p* < 0.01) (Figure 4). There was no significant difference in the risk of hyperkalemia between the combination and Finerenone alone.

According to GRADE assessments (Supplementary Table S5), the certainty of evidence was moderate for all-cause mortality and UACR reduction and low for MACE and MAKE. The risk of hyperkalemia was supported by very low certainty evidence.

TSA was conducted to evaluate the robustness of the pooled effects comparing combination therapy with either finerenone or SGLT2i monotherapy. For all-cause mortality, MACE, and MAKE, the cumulative Z curves did not cross the trial sequential monitoring boundaries for benefit nor enter the futility area (Supplementary Figures S7, S8). Further adequately powered randomized trials are needed to confirm or refute these findings.

## Discussion

This systematic review and meta-analysis evaluated the effects of combined finerenone and SGLT2i therapy in patients with CKD. Compared with finerenone alone, the combination was associated with lower risks of all-cause mortality, MACE, and MAKE. In addition, the combination therapy resulted in a greater reduction in UACR compared to finerenone alone. However, no significant difference in these risks was observed when compared with SGLT2i monotherapy. The risk of hyperkalemia with combination therapy was higher than with SGLT2i alone.

The mortality estimate from Chuang et al. was closer to the null than that from FIDELITY, which may reflect differences between retrospective real-world data and randomized trial populations, including residual confounding, treatment-selection bias, broader CKD case-mix, and potential variability in exposure ascertainment and adherence. In addition, the CONFIDENCE trial contributed no mortality events and had negligible weight in the pooled analysis.

Finerenone reduces oxidative stress, inflammation, and fibrosis through non-hemodynamic pathways, while SGLT2is primarily improve glomerular hemodynamics, metabolic profiles, and tubuloglomerular feedback (Provenzano et al., 2022; Chen J. Y. et al., 2023; Luft, 2021). These agents act through distinct mechanisms that are biologically complementary. Preclinical models have demonstrated that their combination leads to enhanced survival and greater reductions in cardiovascular damage compared to either agent alone, suggesting a synergistic cardioprotective effect (Kolkhof et al., 2021). This synergistic interaction may contribute to the observed reduction in MACE with combination therapy in our analysis.

In addition to cardiovascular benefits, the combination therapy may offer additive renal protection. Both finerenone and SGLT2i independently reduce albuminuria, which has been recognized as a surrogate marker for kidney disease progression (Provenzano et al., 2022; Wanner et al., 2024). Importantly, our meta-analysis showed that combination therapy resulted in a significantly greater reduction in UACR compared with finerenone monotherapy. Although combination therapy resulted in a greater reduction in UACR, substantial heterogeneity was observed across studies (I^2^ = 99.9%). This variability is likely driven by both clinical and methodological differences. From a clinical perspective, there was considerable heterogeneity in baseline albuminuria levels across studies, with UACR ranging from as low as 30 mg/g to as high as 5000 mg/g. Such wide variation may influence the magnitude of treatment response due to potential ceiling effects and regression to the mean. In addition, differences in CKD severity, as reflected by varying eGFR inclusion criteria (ranging from 25 to 90 mL/min/1.73 m^2^), may further contribute to differential responsiveness to therapy. Study design also varied substantially, with both randomized controlled trials and observational cohort studies included. Observational studies may introduce residual confounding and treatment selection bias, potentially amplifying between-study variability. Furthermore, follow-up duration differed across studies, particularly with the relatively short duration in the CONFIDENCE trial (180 days), which may preferentially capture early hemodynamic changes in UACR rather than longer-term structural effects. From a methodological perspective, UACR outcomes were derived from pre–post changes rather than direct between-group comparisons. This approach does not account for within-subject correlation and may be sensitive to baseline imbalances. In addition, heterogeneity in reporting formats (e.g., percentage reduction, absolute change, or potentially log-transformed values) may further contribute to the observed inconsistency. Therefore, while the direction of effect suggests a potential additive benefit of combination therapy on albuminuria reduction, the magnitude of the effect should be interpreted with caution.

Compared with SGLT2i monotherapy, however, combination therapy did not show additional benefits in terms of mortality, MACE, or MAKE. One possible explanation is that SGLT2is already provide substantial renoprotective effects through multiple mechanisms, including reductions in intraglomerular pressure and improvements in tubuloglomerular feedback (Bailey et al., 2022). As a result, the observable incremental benefit of adding finerenone may be limited. In addition, partial overlap in anti-inflammatory and antifibrotic pathways between SGLT2i and finerenone may attenuate the magnitude of additive effects (Bakris et al., 2020; Chen X. et al., 2023). Furthermore, the relatively short follow-up duration and low event rates in some included studies may have reduced the ability to detect modest additional benefits of combination therapy.

Importantly, SGLT2is have also been shown to reduce the risk of hyperkalemia, potentially through kaliuretic effects mediated by increased distal sodium delivery and urine flow, as well as elevated glucagon levels (Palmer and Clegg, 2024). In contrast, finerenone, as a mineralocorticoid receptor antagonist, is associated with impaired potassium excretion, particularly in patients with reduced eGFR (Bakris et al., 2023). From a clinical perspective, the risk of hyperkalemia should be interpreted primarily in comparison with finerenone monotherapy as mineralocorticoid receptor antagonism represents the dominant driver of potassium retention. Although SGLT2i may exert a potassium-lowering effect, this effect may not be sufficient to fully counterbalance the aldosterone-mediated reduction in distal potassium secretion induced by finerenone (Agarwal et al., 2026; Agarwal et al., 2022). In addition, in patients with CKD, impaired distal tubular function may attenuate the kaliuretic response to SGLT2i, thereby limiting their protective effect (Palmer and Clegg, 2024). These mechanisms may explain why combination therapy did not demonstrate a meaningful reduction in hyperkalemia risk compared with finerenone alone. These differing effects on potassium regulation may help explain the numerically higher risk of hyperkalemia observed with combination therapy compared to SGLT2i alone.

To our knowledge, this is the first meta-analysis following the CONFIDENCE trial (Agarwal et al., 2025) to directly compare combined therapy with SGLT2i and finerenone against monotherapy with either agent. By integrating data from both RCTs and observational studies, this study provides comprehensive evidence on the effects of combination therapy *versus* monotherapy on mortality, cardiovascular, and kidney outcomes, including albuminuria reduction, offering valuable insights to inform future clinical decision-making.

However, this analysis has several limitations. First, for FIDELIO-DKD (Bakris et al., 2020) and FIGARO-DKD (Pitt et al., 2021), outcome data for combination therapy were derived from subgroup analyses. Therefore, baseline characteristics such as age and sex were extracted from the overall study population, which may include individuals who were not part of the selected subgroups, potentially limiting the precision of population-level interpretation. Second, many of the included studies were finerenone trials in which SGLT2i use was retrospectively identified to define the combination therapy group, which may introduce potential information bias. Third, the inclusion of multiple SGLT2i with varying doses, compared with a single MRA (finerenone), may introduce clinical heterogeneity and affect the comparability of treatment effects. Fourth, the CONFIDENCE trial had a relatively short follow-up duration of only 180 days (Agarwal et al., 2025), which may have resulted in underascertainment of long-term hard outcomes and introduced a form of detection bias. Fifth, for the analysis of UACR reduction, we extracted and compared the mean changes from baseline to follow-up within each group. This approach, while commonly used, reflects a difference in pre-post changes rather than a direct between-group comparison and does not account for the within-subject correlation between baseline and post-treatment measurements. As noted in previous methodological research, such pre–post effect size estimations may introduce bias due to natural disease progression and uncontrolled confounding factors (Cuijpers et al., 2017). Therefore, findings regarding UACR reduction should be interpreted with caution.

## Conclusion

In summary, this meta-analysis suggests that the combined use of finerenone and SGLT2i offers greater cardiorenal protection than finerenone alone, as evidenced by reductions in mortality, MACEs, MAKEs, and UACR. However, the combination did not show additional benefit compared to SGLT2i monotherapy and was associated with an increased risk of hyperkalemia. These findings highlight both the potential advantages and safety considerations of combination therapy, supporting the need for individualized treatment decisions and further evaluation.

## Data Availability

The original contributions presented in the study are included in the article/Supplementary Material, further inquiries can be directed to the corresponding author.
