# Correction: Efficacy of combined sodium-glucose cotransporter 2 inhibitors and finerenone in chronic kidney disease: a systematic review and meta-analysis

**DOI:** 10.3389/fphar.2026.1902375

**Published:** 2026-09-08

**Authors:** Chen-Fu Wen, Min-Hsiang Chuang, Vin-Cent Wu, Jui-Yi Chen

**Affiliations:** 1 Department of Internal Medicine, Chi Mei Medical Center, Tainan, Taiwan; 2 Division of Nephrology, Department of Internal Medicine, Chi Mei Medical Center, Tainan, Taiwan; 3 Division of Nephrology, Department of Internal Medicine, National Taiwan University Hospital, Tainan, Taiwan; 4 Department of Health and Nutrition, Chia Nan University of Pharmacy and Science, Tainan, Taiwan; 5 Department of Public Health, College of Medicine, National Cheng Kung University, Tainan, Taiwan

**Keywords:** cardiovascular outcome, chronic kidney disease, finerenone, hyperkalemia, meta-analysis, sodium-glucose cotransporter 2 inhibitor

Author Chen-Fu Wen was erroneously assigned as corresponding author. The correct corresponding author is Jui-Yi Chen.

The original version of this article has been updated.

