# Supplementary material for: Efficacy of combined sodium-glucose cotransporter 2 inhibitors and finerenone in chronic kidney disease: a systematic review and meta-analysis
Source: Front Pharmacol. 2026 May 13;17:1803971. doi: 10.3389/fphar.2026.1803971 (PMC13212455; doi:10.3389/fphar.2026.1803971)
Supplement: Supplementary file 1 [file Supplementaryfile1.docx]

**Supplementary appendix**

**Efficacy of Combined Sodium-Glucose Cotransporter 2 Inhibitors and Finerenone in Chronic Kidney Disease: A Systematic Review and Meta-Analysis**

Authors:

Chen-Fu Wen, Min-Hsiang Chuang, Vin-Cent Wu, Jui-Yi Chen

This supplementary appendix provides:

1. Table S1. PROSPERO protocol registration
2. Table S2. 2020 PRISMA checklist
3. Table S3. Search strategies for PubMed, EMBASE and Cochrane library
4. Table S4. Outcome contribution by study
5. Table S5. The GRADE of all cause mortality, MACE, MAKE, hyperkalemia
6. Figure S1. The quality assessment for the included studies in our meta-analysis using version 2 of the Cochrane risk of bias tool
7. Figure S2. Forest plots comparing combined therapy versus SGLT2i monotherapy for (a) all-cause mortality, (b) MACE, and (c) MAKE
8. Figure S3. Funnel plots showing a potential risk of bias on the combined therapy versus finerenone monotherapy
9. Figure S4. Funnel plots showing a potential risk of bias on the combined therapy versus SGLT2i monotherapy
10. Figure S5. Sensitivity analysis of excluding observational study comparing combined therapy versus Finerenone monotherapy
11. Figure S6. Sensitivity analysis of excluding observational study comparing combined therapy versus SGLT2i monotherapy
12. Figure S7. Trial sequential analysis showing a potential risk of bias on the combined therapy versus Finerenone monotherapy
13. Figure S8. Trial sequential analysis showing a potential risk of bias on the combined therapy versus SGLT2i monotherapy

**Table S1. PROSPERO protocol registration**

REVIEW TITLE AND BASIC DETAILS

- Review title

Combined SGLT2i and Finerenone Therapy in Diabetic CKD: A Meta-Analysis

- Condition or domain being studied

Diabetes Mellitus; Chronic Kidney Disease; Sodium-glucose Co-transporter 2 (SGLT2) Inhibitors; Finerenone

- Rationale for the review

This meta-analysis was conducted to clarify the efficacy of combined SGLT2i and finerenone therapy in CKD and T2DM patients. While both drugs show individual benefits, evidence on their combined impact on mortality, MACE, and MAKE remains inconclusive, necessitating a systematic evaluation of their potential synergistic effects.

- Review objectives

Does combination therapy provide superior benefits compared to monotherapy with either SGLT2i or finerenone in reducing mortality?

- Keywords

Finerenone; SGLT2 inhibitors; Diabetes kidney disease; Major adverse cardiovascular events MACE; Major adverse kidney event; All-cause mortality

- Country

Taiwan, Province of China

ELIGIBILITY CRITERIA

- Population

Included: Adults with diabetes mellitus and chronic kidney disease

Excluded: Adolescents (under 18 years of age), End stage renal disease under hemodialysis or peritoneal dialysis, Pregnant women

- Intervention(s) or exposure(s)

Included: Sodium-glucose Co-transporter 2 (SGLT2) Inhibitors; Finerenone Intervention is combination therapy of finerenone with any sodium-glucose cotransporter 2 (SGLT2) inhibitor

- Comparator(s) or control(s)

Included PICO tags selected: Sodium-glucose Co-transporter 2 (SGLT2) Inhibitors; Finerenone

Comparators is monotherapy with either finerenone or any SGLT2 inhibitor

- Study design

Both randomized and nonrandomized study types will be included.

Included: randomized controlled trials (RCTs) and observational studies

Excluded: 1.trials that included animals or pregnant women 2.research designs not assess death, MACE, MAKE, such as those focused on atrial fibrillation risk 3.studies focusing on populations with dialysis-dependent kidney failure or kidney transplant recipients.

- Context

trials that included animals or pregnant women would be excluded

TIMELINE OF THE REVIEW

- Date of first submission to PROSPERO

05 April 2025

- Review timeline

Start date: 24 February 2025. End date: 06 June 2025.

- Date of registration in PROSPERO

20 May 2025

AVAILABILITY OF FULL PROTOCOL

- Availability of full protocol

A full protocol has not been written.

SEARCHING AND SCREENING

- Search for unpublished studies

Only published studies will be sought.

- Main bibliographic databases that will be searched

The main databases to be searched are CLIB - The Cochrane Library, Embase - Embase via Ovid and PubMed.

- Search language restrictions

There are no language restrictions.

- Search date restrictions

There are no search date restrictions.

- Other methods of identifying studies

Other studies will be identified by: contacting authors or experts, looking through all the articles that cite the papers included in the review ("snowballing") and reference list checking.

- Link to search strategy

A full search strategy is available in the full protocol as described in the Availability of full protocol section

- Selection process

Studies will be screened by one person (or a machine) and checked by at least one other person (or machine).

- Other relevant information about searching and screening

None

DATA COLLECTION PROCESS

- Data extraction from published articles and reports

Data will be extracted independently by at least two people (or person/machine combination) with a process to resolve differences.

Authors will be asked to provide any required data not available in published reports.

Study datasets/IPD will be obtained from study investigators or via a data repository

- Study risk of bias or quality assessment

Risk of bias will be assessed using: Cochrane RoB-2

Data will be assessed by one person (or a machine) and checked by at least one other person (or machine).

Additional information will be sought from study investigators if required information is unclear or unavailable in the study publications/reports.

- Reporting bias assessment

Risk of bias due to missing results will be assessed

- Certainty assessment

funnel plot will be used to assess certainty

OUTCOMES TO BE ANALYSED

- Main outcomes

assess any of the following: death from any cause, major adverse cardiovascular event(MACE), major adverse kidney event(MAKE)

- Additional outcomes

There are no additional outcomes.

PLANNED DATA SYNTHESIS

- Strategy for data synthesis

Comprehensive Meta-Analysis software, version 3 (Biostat, Englewood, NJ, USA) was used for all statistical analyses. A P-value < 0.05 was deemed statistically significant. For continuous outcomes, the results were expressed as standardized mean difference (SMD) with 95% confidence interval (CI). Dichotomous outcomes were presented as odds ratios (ORs) with their associated 95% CIs.

CURRENT REVIEW STAGE

- Stage of the review at this submission
- Review stage

Pilot work : completed

Formal searching/study identification : completed

Screening search results against inclusion criteria : completed

Data extraction or receipt of IPD : completed

Risk of bias/quality assessment : completed

Data synthesis : completed

- Review status : completed

The review is currently planned or ongoing.

- Publication of review results

Results of the review will be published in English

REVIEW AFFILIATION, FUNDING AND PEER REVIEW

- Review team members

Mr Chen Fu Wen (review guarantor and contact) Chi Mei Medical Center. Taiwan. No conflict of interest declared.

Mr Chen Jui Yi. Chi Mei Medical Center. Taiwan. No conflict of interest declared.

- Named contact

Mr Chen Fu Wen (violencemonster@gmail.com). Chi Mei Medical Center. Taiwan.

- Review affiliation

Chi Mei Medical Center, Tainan, Taiwan

- Funding source

Review has no specific/external funding but is supported by guarantor/review team (non commercial) institutions.

- Peer review

There has been no peer review of this planned review.

ADDITIONAL INFORMATION

- Review conflict of interest

Declared individual interests are recorded under team member details.. No additional interests are recorded for this review.

- Medical Subject Headings

Diabetes Mellitus; Renal Insufficiency, Chronic; Sodium-Glucose Transporter 2 Inhibitors; finerenone

SIMILAR REVIEWS

- Check for similar records already in PROSPERO

PROSPERO identified a number of existing PROSPERO records that were similar to this one (last check made on 4 April 2025). These are shown below along with the reasons given by that the review team for the reviews being different and/or proceeding.

*Dual Therapy with SGLT2 Inhibitors and MRAs vs. Monotherapy in Heart Failure: A Systematic Review and Meta-Analysis [published 24 February 2025] [CRD420250656366].*

The review was judged not to be similar

*Efficacy of SGLT-2 Inhibitors combined with Non-Steroidal Mineralocorticoid Receptor Antagonists in patients with cardiovascular-kidney-metabolic diseases and their impact on cardiorenal outcomes: A systematic review and meta-Analysis [published 30 March 2025] [CRD420251022349].*

The review was judged not to be similar

*Efficacy and Safety of SGLT2 Inhibitors and Finerenone in Diabetic kidney disease and Non-Diabetic Chronic Kidney Disease (FIND-CKD): A Meta-Analysis [published 23 March 2025] [CRD420251017518].*

The review was judged not to be similar

**Table S2. 2020 PRISMA checklist**

| **Section and Topic** | **Item #** | **Checklist item** | **Location where item is reported** |
| --- | --- | --- | --- |
| **TITLE** | | |  |
| Title | 1 | Identify the report as a systematic review. | Title includes "Systematic Review and Meta-Analysis" |
| **ABSTRACT** | | |  |
| Abstract | 2 | See the PRISMA 2020 for Abstracts checklist. | Structured abstract includes background, methods, results, and conclusions |
| **INTRODUCTION** | | |  |
| Rationale | 3 | Describe the rationale for the review in the context of existing knowledge. | Provided in Introduction |
| Objectives | 4 | Provide an explicit statement of the objective(s) or question(s) the review addresses. | To evaluate whether combined therapy with SGLT2i and finerenone improves renal and cardiovascular outcomes |
| **METHODS** | | |  |
| Eligibility criteria | 5 | Specify the inclusion and exclusion criteria for the review and how studies were grouped for the syntheses. | Stated in Methods section |
| Information sources | 6 | Specify all databases, registers, websites, organisations, reference lists and other sources searched or consulted to identify studies. Specify the date when each source was last searched or consulted. | PubMed, Embase, Cochrane; searched up to June 6, 2025 |
| Search strategy | 7 | Present the full search strategies for all databases, registers and websites, including any filters and limits used. | Full strategies are in supplement Table S1 |
| Selection process | 8 | Specify the methods used to decide whether a study met the inclusion criteria of the review, including how many reviewers screened each record and each report retrieved, whether they worked independently, and if applicable, details of automation tools used in the process. | Two independent reviewers; consensus resolution |
| Data collection process | 9 | Specify the methods used to collect data from reports, including how many reviewers collected data from each report, whether they worked independently, any processes for obtaining or confirming data from study investigators, and if applicable, details of automation tools used in the process. | Standardized form used by two independent reviewers |
| Data items | 10a | List and define all outcomes for which data were sought. Specify whether all results that were compatible with each outcome domain in each study were sought (e.g. for all measures, time points, analyses), and if not, the methods used to decide which results to collect. | All-caused mortality, MACE, MAKE, hyperkalemia |
|  | 10b | List and define all other variables for which data were sought (e.g. participant and intervention characteristics, funding sources). Describe any assumptions made about any missing or unclear information. | Population, intervention, study design, follow-up, which kind of SGLT2i and the dosage |
| Study risk of bias assessment | 11 | Specify the methods used to assess risk of bias in the included studies, including details of the tool(s) used, how many reviewers assessed each study and whether they worked independently, and if applicable, details of automation tools used in the process. | ROB 2.0 (RCT and post-hoc analysis), ROBINS-I (observational) |
| Effect measures | 12 | Specify for each outcome the effect measure(s) (e.g. risk ratio, mean difference) used in the synthesis or presentation of results. | Odds ratios (OR) with 95% CI |
| Synthesis methods | 13a | Describe the processes used to decide which studies were eligible for each synthesis (e.g. tabulating the study intervention characteristics and comparing against the planned groups for each synthesis (item #5)). | Random-effects meta-analysis, subgroup, sensitivity, TSA analysis |
|  | 13b | Describe any methods required to prepare the data for presentation or synthesis, such as handling of missing summary statistics, or data conversions. | Funnel plots |
|  | 13c | Describe any methods used to tabulate or visually display results of individual studies and syntheses. | Table and Forrest plot |
|  | 13d | Describe any methods used to synthesize results and provide a rationale for the choice(s). If meta-analysis was performed, describe the model(s), method(s) to identify the presence and extent of statistical heterogeneity, and software package(s) used. | Forrest plot |
|  | 13e | Describe any methods used to explore possible causes of heterogeneity among study results (e.g. subgroup analysis, meta-regression). | subgroup analysis |
|  | 13f | Describe any sensitivity analyses conducted to assess robustness of the synthesized results. | Trial sequential analysis |
| Reporting bias assessment | 14 | Describe any methods used to assess risk of bias due to missing results in a synthesis (arising from reporting biases). | Funnel plots |
| Certainty assessment | 15 | Describe any methods used to assess certainty (or confidence) in the body of evidence for an outcome. | GRADE used for outcome certainty |
| **RESULTS** | | |  |
| Study selection | 16a | Describe the results of the search and selection process, from the number of records identified in the search to the number of studies included in the review, ideally using a flow diagram. | PRISMA diagram included |
|  | 16b | Cite studies that might appear to meet the inclusion criteria, but which were excluded, and explain why they were excluded. | The last part of PRISMA flow diagram show reasons of studies excluded. |
| Study characteristics | 17 | Cite each included study and present its characteristics. | Included 8 studies (4 RCTs, 1post-hoc analysis, 3 observational study) |
| Risk of bias in studies | 18 | Present assessments of risk of bias for each included study. | RoB 2 and ROBINS-I assessments presented in figures and tables. |
| Results of individual studies | 19 | For all outcomes, present, for each study: (a) summary statistics for each group (where appropriate) and (b) an effect estimate and its precision (e.g. confidence/credible interval), ideally using structured tables or plots. | Individual results presented in summary tables with effect estimates and confidence intervals. |
| Results of syntheses | 20a | For each synthesis, briefly summarise the characteristics and risk of bias among contributing studies. | Results presented in figure with effect estimates and confidence intervals. |
|  | 20b | Present results of all statistical syntheses conducted. If meta-analysis was done, present for each the summary estimate and its precision (e.g. confidence/credible interval) and measures of statistical heterogeneity. If comparing groups, describe the direction of the effect. | Results presented in figure with effect estimates and confidence intervals |
|  | 20c | Present results of all investigations of possible causes of heterogeneity among study results. | Results presented in figure with effect estimates and confidence intervals |
|  | 20d | Present results of all sensitivity analyses conducted to assess the robustness of the synthesized results. | Results presented in figure with effect estimates and confidence intervals |
| Reporting biases | 21 | Present assessments of risk of bias due to missing results (arising from reporting biases) for each synthesis assessed. | Funnel plot appears symmetrical |
| Certainty of evidence | 22 | Present assessments of certainty (or confidence) in the body of evidence for each outcome assessed. | GRADE: moderate-to-high certainty for renal outcomes, low certainty for cardiovascular outcomes due to imprecision. |
| **DISCUSSION** | | |  |
| Discussion | 23a | Provide a general interpretation of the results in the context of other evidence. | Combined use of SGLT2 inhibitors and finerenone appears to improve renal outcomes in diabetic CKD; cardiovascular benefits less certain. |
|  | 23b | Discuss any limitations of the evidence included in the review. | Limited number of studies; variation in baseline characteristics; some included observational designs. |
|  | 23c | Discuss any limitations of the review processes used. | Limited to English language, some unpublished data not retrievable |
|  | 23d | Discuss implications of the results for practice, policy, and future research. | Findings support potential additive benefit. Further large-scale RCTs are needed to confirm long-term outcomes. |
| **OTHER INFORMATION** | | |  |
| Registration and protocol | 24a | Provide registration information for the review, including register name and registration number, or state that the review was not registered. | Registered in PROSPERO (ID: CRD420251023918); protocol available upon request. |
|  | 24b | Indicate where the review protocol can be accessed, or state that a protocol was not prepared. | Registered in PROSPERO (ID: CRD420251023918); protocol available upon request |
|  | 24c | Describe and explain any amendments to information provided at registration or in the protocol. | Registered in PROSPERO (ID: CRD420251023918); protocol available upon request |
| Support | 25 | Describe sources of financial or non-financial support for the review, and the role of the funders or sponsors in the review. | No specific funding received for this review. |
| Competing interests | 26 | Declare any competing interests of review authors. | Authors declare no competing interests. |
| Availability of data, code and other materials | 27 | Report which of the following are publicly available and where they can be found: template data collection forms; data extracted from included studies; data used for all analyses; analytic code; any other materials used in the review. | All data and code used in this review are available upon reasonable request. |

**Table S3. Search Strategy.**

**Search strategies for PubMed, EMBASE and Cochrane library**

**Appendix.**

**Search strategies for the different databases ran on June 6, 2025**

**PubMed Search Query**

(("sodium glucose transporter 2 inhibitors"[Pharmacological Action] OR "sodium glucose transporter 2 inhibitors"[Supplementary Concept] OR "sodium glucose transporter 2 inhibitors"[All Fields] OR "sodium glucose transporter 2 inhibitors"[All Fields] OR "sodium glucose transporter 2 inhibitors"[MeSH Terms] OR ("finerenone"[Supplementary Concept] OR "finerenone"[All Fields])) AND ("renal insufficiency, chronic"[MeSH Terms] OR ("renal"[All Fields] AND "insufficiency"[All Fields] AND "chronic"[All Fields]) OR "chronic renal insufficiency"[All Fields] OR ("chronic"[All Fields] AND "kidney"[All Fields] AND "disease"[All Fields]) OR "chronic kidney disease"[All Fields])) AND ((clinicaltrial[Filter] OR observationalstudy[Filter] OR randomizedcontrolledtrial[Filter]) AND (fft[Filter]))

Filters: Full text, Observational Study, Randomized Controlled Trial

Result: 278

**EMBASE**

(exp sodium glucose cotransporter 2 inhibitor/ OR sodium glucose cotransporter 2 inhibitor.tw. OR sglt2i.tw. OR dapagliflozin.tw. OR empagliflozin.tw. OR canagliflozin.tw. OR ertugliflozin.tw. OR exp finerenone/ OR finerenone.tw.)

AND

(exp chronic kidney disease/ OR chronic kidney disease.tw. OR renal insufficiency chronic.tw. OR chronic renal insufficiency.tw.)

AND

(clinical trial.pt. OR observational study.pt. OR randomized controlled trial.pt.)

AND

embase.db.

Filters: Research articles

Result: 246

**Cochrane Library**

("sodium glucose cotransporter 2 inhibitor" OR sglt2i OR dapagliflozin OR empagliflozin OR canagliflozin OR ertugliflozin OR finerenone)

AND

("chronic kidney disease" OR "chronic renal insufficiency" OR "renal insufficiency, chronic")

Result: 1056

**Table S4. Outcome contribution by study**

| **Outcome** | **Dataset used** | **Combine*#** | **Finerenone*** | **SGLT2i*** |
| --- | --- | --- | --- | --- |
| All-cause mortality | FIDELITY + Chuang + CONFIDENCE  **Total** | 20/438+  11/801 or 16/1335  +0/268=  **31/1507 or 36/2041** | 532/6,081+  13/801+  0/264=  **545/7146** | 30/439+  19/1335+  0/266=  **49/2040** |
| MACE | FIDELIO-DKD + FIGARO-DKD + Chuang + CONFIDENCE  **Total** | 15/124+  24/314+  16/801 or 25/1335+  2/268=  **57/1507 or 65/2041** | 352/2,709+ 434/3,372+ 22/801+  0/264=  **808/7146** | 15/135+  37/304+  30/1335+  1/266=  **83/2040** |
| MAKE | FIDELIO-DKD + FIGARO-DKD + Chuang + CONFIDENCE  **Total** | 14/124+  6/314+  24/801 or 35/1335  +0/268=  **44/1507 or 55/2041** | 490/2,709+  102/3,372+  34/801+  2/264=  **628/7146** | 10/135+  11/304+  42/1335+  1/266=  **64/2040** |
| UACR reduction | FIDELITY + Hanouneh + Mårup + CONFIDENCE + Kovesdy |  |  |  |
| Hyperkalemia | FIDELITY + Chuang + CONFIDENCE  **Total** | 45/438+  299/2,298 or 387/3,407  +25/268=  **369/3004 or 457/4113** | 867/6,072+  147/2,298+  30/264=  **1044/8634** | 12/439+  142/3,407+  10/266=  **164/4112** |

**Abbreviations:** MACE, major adverse cardiovascular event; MAKE, major adverse kidney event; UACR, Urinary albumin creatinine ratio

Abbreviations: MACE, major adverse cardiovascular event; MAKE, major adverse kidney event; UACR, Urinary albumin creatinine ratio

**Table S5. The GRADE of all-cause mortality, MACE, MAKE, hyperkalemia, UACR reduction**

| Certainty assessment | | | | | | | Study event rate | | | Effect  (Com v.s. S) | Effect  (Com v.s. F) | Quality of evidence |
| --- | --- | --- | --- | --- | --- | --- | --- | --- | --- | --- | --- | --- |
| No of studies | Study design(number) | Risk of bias | Inconsistency | Indirectness | Imprecision | Publication bias | Combine | SGLT2i | Finerenone | OR  (95% CI) | OR  (95% CI) |  |
| All-cause mortality | | | | | | | | | | | | |
| 3 | RCT (1), Post-hoc analysis (1), Observational study (1) | not serious | not serious | not serious | not serious | undetected | 29/1,176  (2.5%) | 46/1,326  (3.5%) | 543/6,819  (8.0%) | 0.73  (0.47 to 1.13) | 0.58  (0.36 to 0.93) | ⊕⊕⊕◯  Moderate |
| MACE | | | | | | | | | | | | |
| 4 | RCT (3),  Observational study (1) | not serious | not serious | not serious | not serious | undetected | 42/1,176  (3.6%) | 63/1,326  (4.8%) | 795/6,819  (11.7%) | 0.77  (0.55 to 1.08) | 0.70  (0.51 to 0.97) | ⊕⊕◯◯  Low |
| MAKE | | | | | | | | | | | | |
| 4 | RCT(3),  Observational study(1) | not serious | not serious | not serious | not serious | undetected | 31/1,176  (2.6%) | 35/1,326  (2.6%) | 604/6,819  (8.9%) | 0.87  (0.58 to 1.31) | 0.63  (0.44 to 0.89) | ⊕⊕◯◯  Low |
| UACR reduction | | | | | | | | | | | | |
| 5 | RCT (2), Post-hoc analysis (1), Observational study (2) | serious | serious | not serious | not serious | undetected |  | NR |  | NR | 0.11  (0.02 to 0.20) | ⊕⊕⊕◯  Moderate |
| hyperkalemia | | | | | | | | | | | | |
| 3 | RCT (1), Post-hoc analysis (1), Observational study (1) | serious (inconsistent reporting in observational study) | not serious | serious (limited clinical correlation) | serious | undetected | 128/1,176  (10.9%) | 72/1,326  (5.4%) | 1,076/6,819  (15.8%) | 3.00  (2.50 to 3.61) | 1.09  (0.52 to 2.28) | ⊕◯◯◯  Very low |

**Abbreviations:** Com, combined; CI, confidence interval; F, finerenone; MACE, major adverse cardiovascular event; MAKE, major adverse kidney event; NR, not reported; OR, odds ratio; RCT, randomized control trial; S, SGLT2i; SGLT2i, sodium-glucose co-transporter 2 inhibitor; UACR, urine albumin-to-creatinine ratio; v.s., versus

**Figure S1A. Risk of bias in the selected studies using the Version 2 of the Cochrane Risk of Bias Tool (ROB 2.0) for RCT**

**
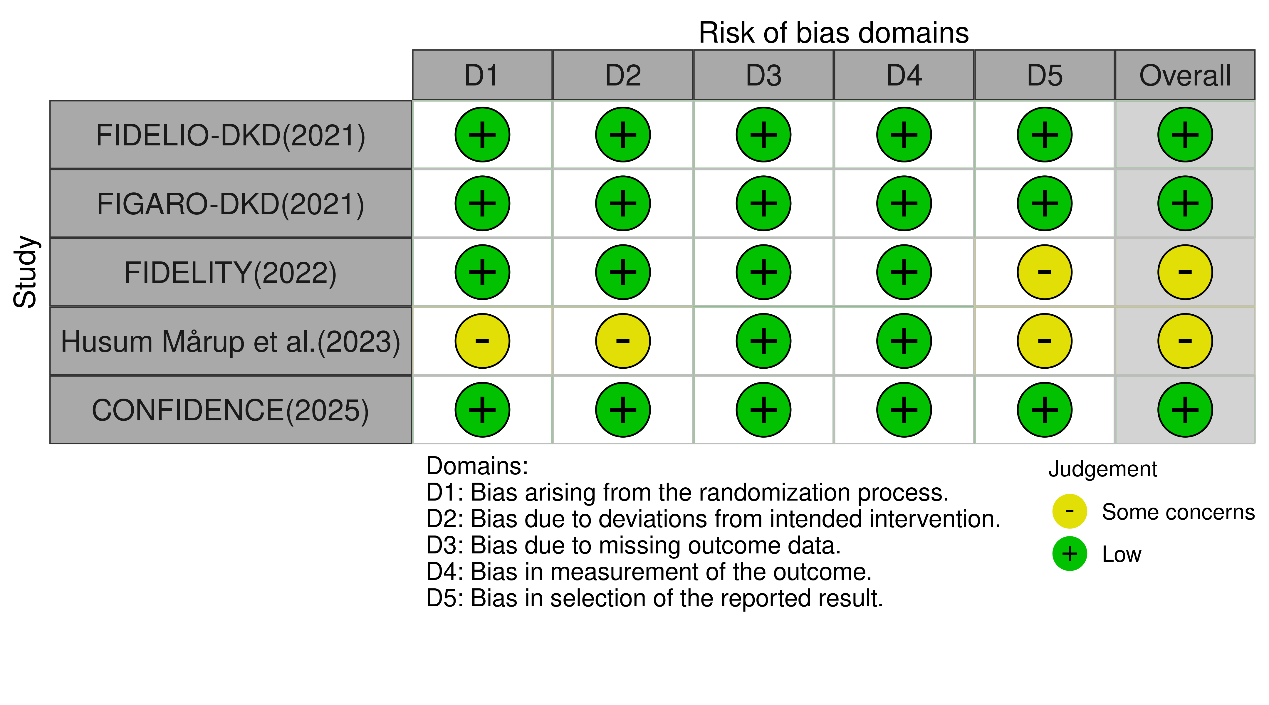
**

**
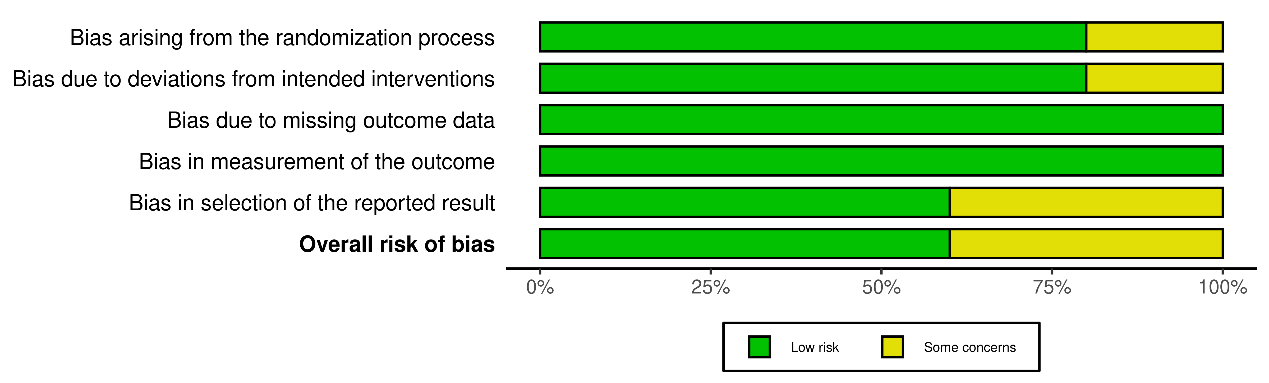
**

**Figure S1B. Risk of bias in the selected studies using the Risk Of Bias In Non-randomized Studies – of Interventions (ROBINS-I) for observational study**

**
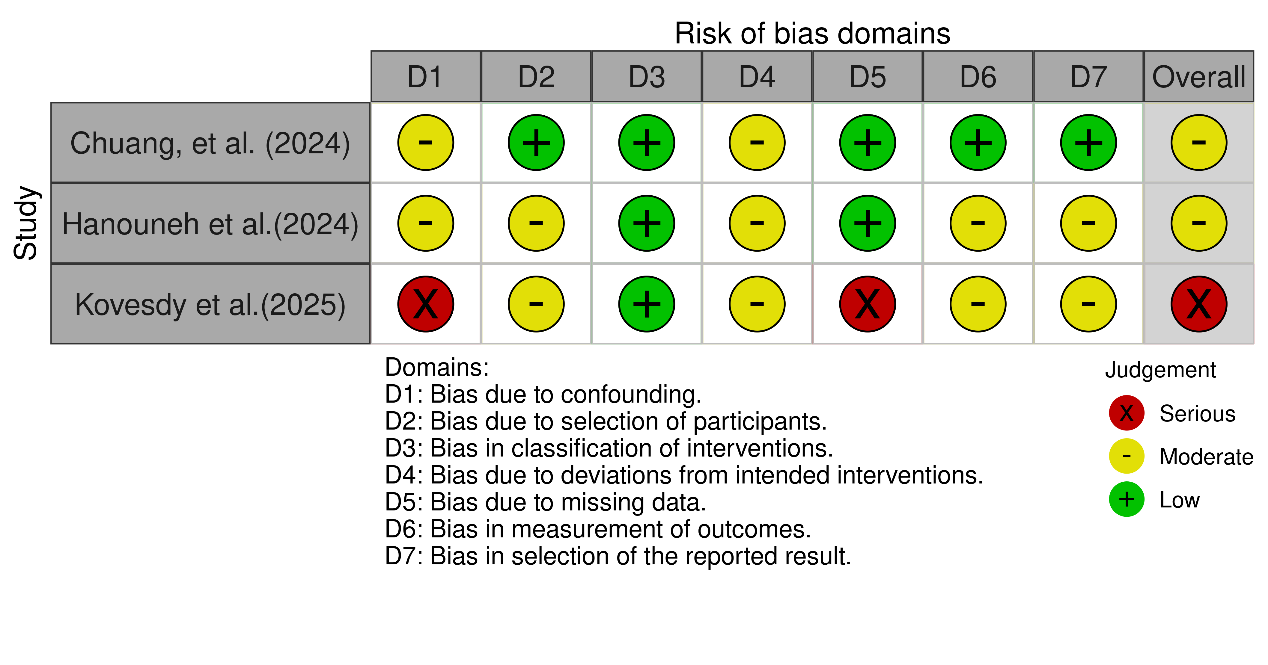
**

**
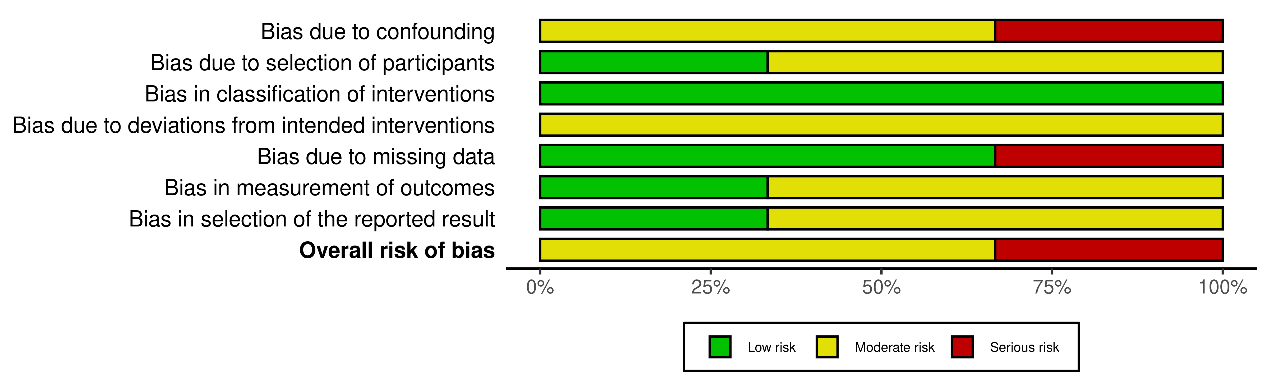
**

**Figure S2. Forest plots comparing combined therapy versus SGLT2i monotherapy for (a) all-cause mortality, (b) MACE, and (c) MAKE**

**
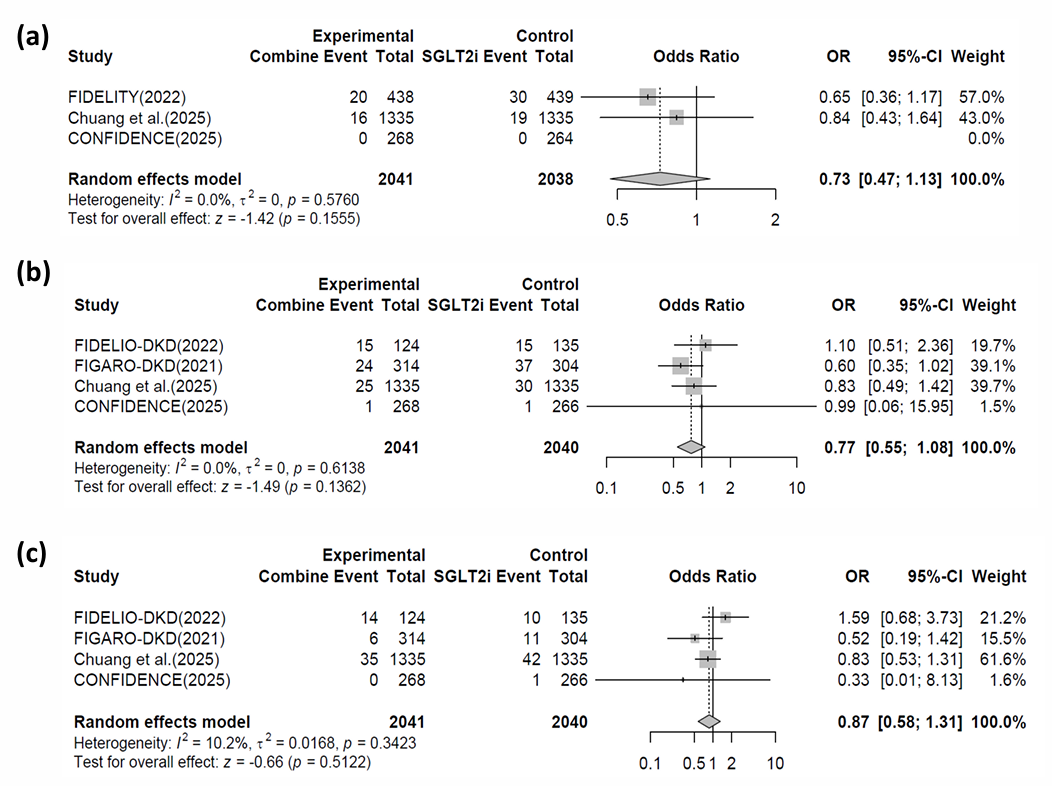
**

**Abbreviations:** CI, confidence interval; MACE, major adverse cardiovascular event; MAKE, major adverse kidney event; OR, odds ratio; SGLT2i, sodium-glucose co-transporter 2 inhibitor

**Figure S3. Funnel plots showing a potential risk of bias on the combined therapy versus Finerenone monotherapy on (a) all-cause mortality, (b) MACE, (c) MAKE**

**(a)**

**
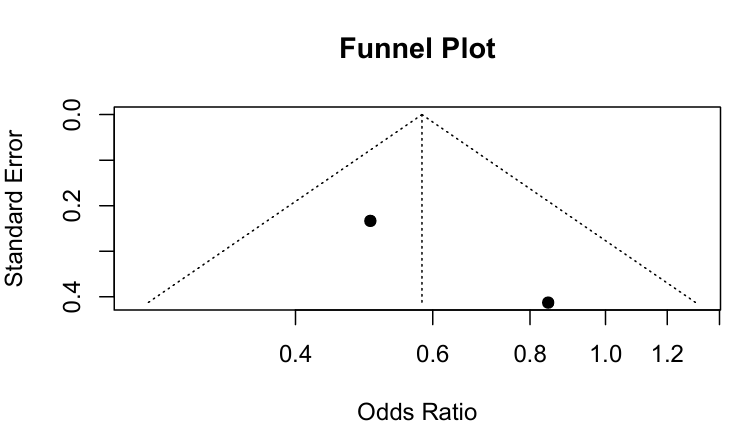
**

**(b)**

**
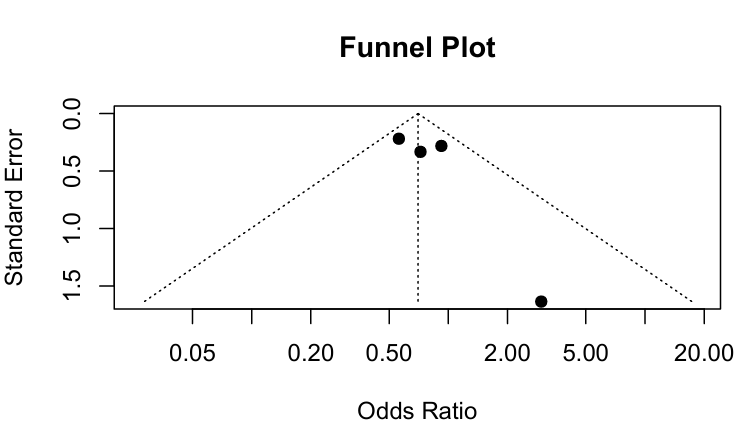
**

**(c)**

**
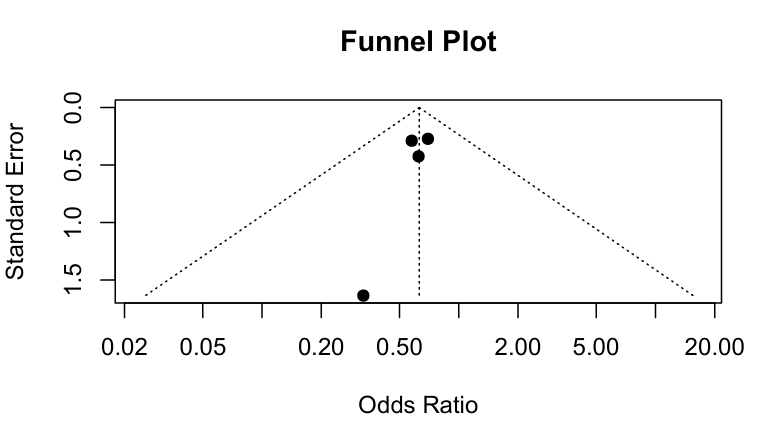
**

**Abbreviations:** MACE, major adverse cardiovascular event; MAKE, major adverse kidney event

**Figure S4 Funnel plots showing a potential risk of bias on the combined therapy versus SGLT2i monotherapy on (a) all-cause mortality, (b) MACE, (c) MAKE**

**(a)**

**
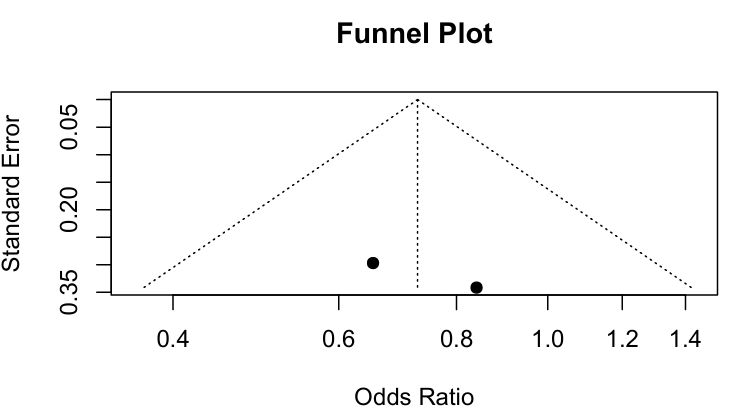
**

**(b)**

**
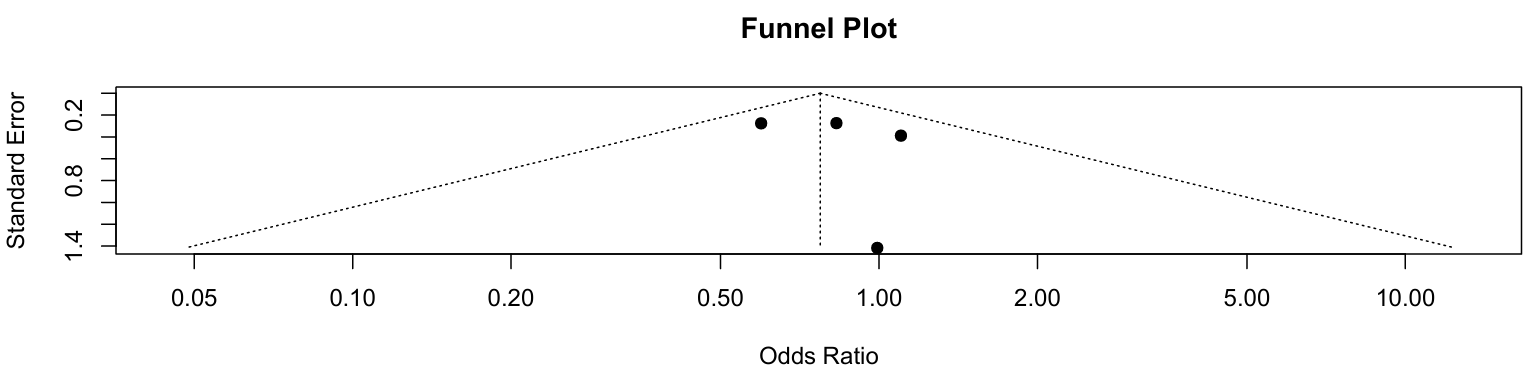
**

**(c)**

**
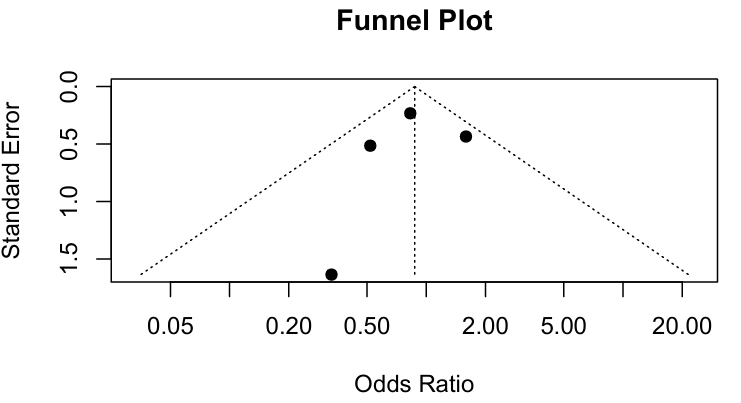
**

**Abbreviations:** MACE, major adverse cardiovascular event; MAKE, major adverse kidney event; SGLT2i, sodium-glucose co-transporter 2 inhibitor

**Figure S5. Sensitivity analysis of excluding observational study comparing combined therapy versus Finerenone monotherapy for (a) MACE and (b) MAKE**


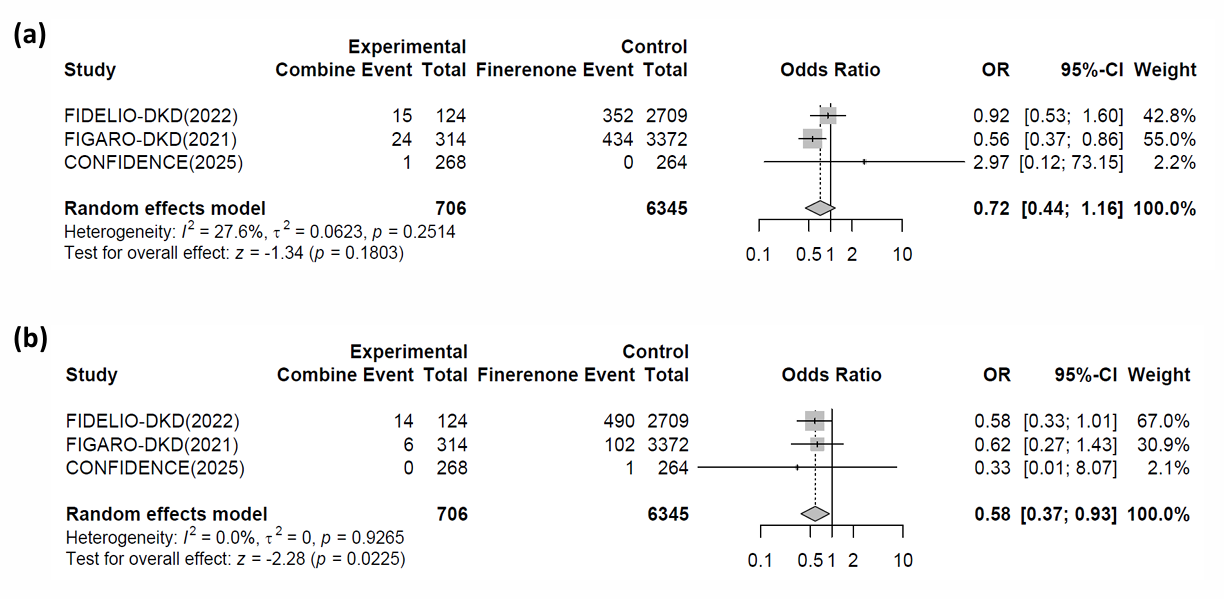


**Abbreviations:** CI, confidence interval; MACE, major adverse cardiovascular event; MAKE, major adverse kidney event; OR, odds ratio; SGLT2i, sodium-glucose co-transporter 2 inhibitor

**Figure S6 Sensitivity analysis of excluding observational study comparing combined therapy versus SGLT2i monotherapy for (a) MACE and (b) MAKE**


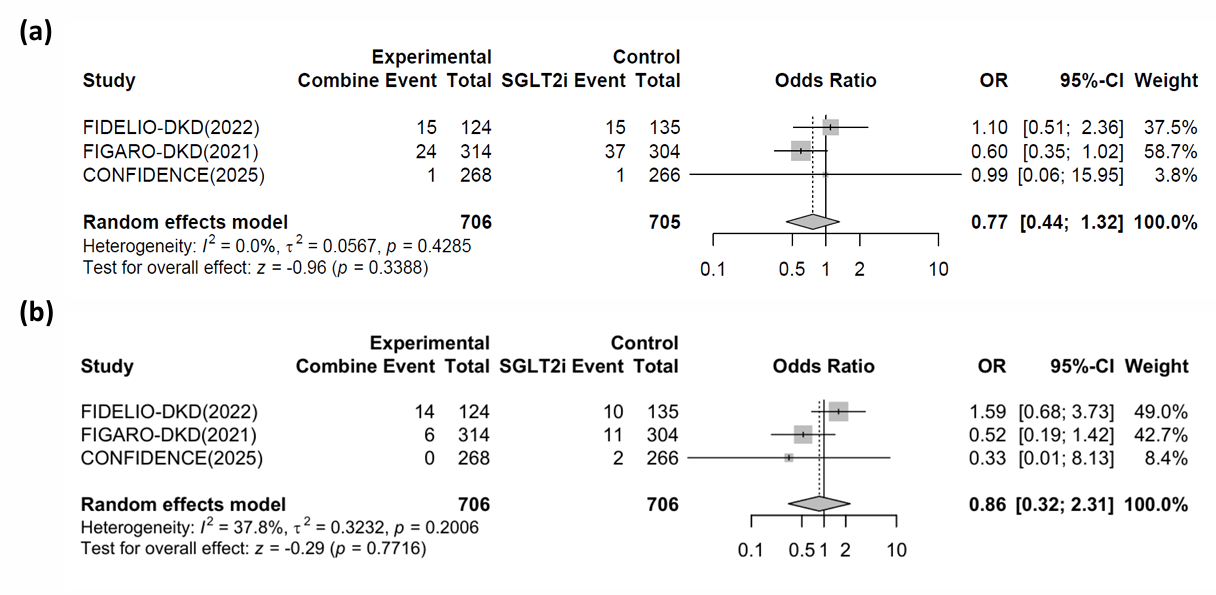


**Abbreviations:** CI, confidence interval; MACE, major adverse cardiovascular event; MAKE, major adverse kidney event; OR, odds ratio; SGLT2i, sodium-glucose co-transporter 2 inhibitor

**Figure S7. Trial sequential analysis showing a potential risk of bias on the combined therapy versus Finerenone monotherapy on (a) all-cause mortality, (b) MACE, (c) MAKE**

**(a)**

**
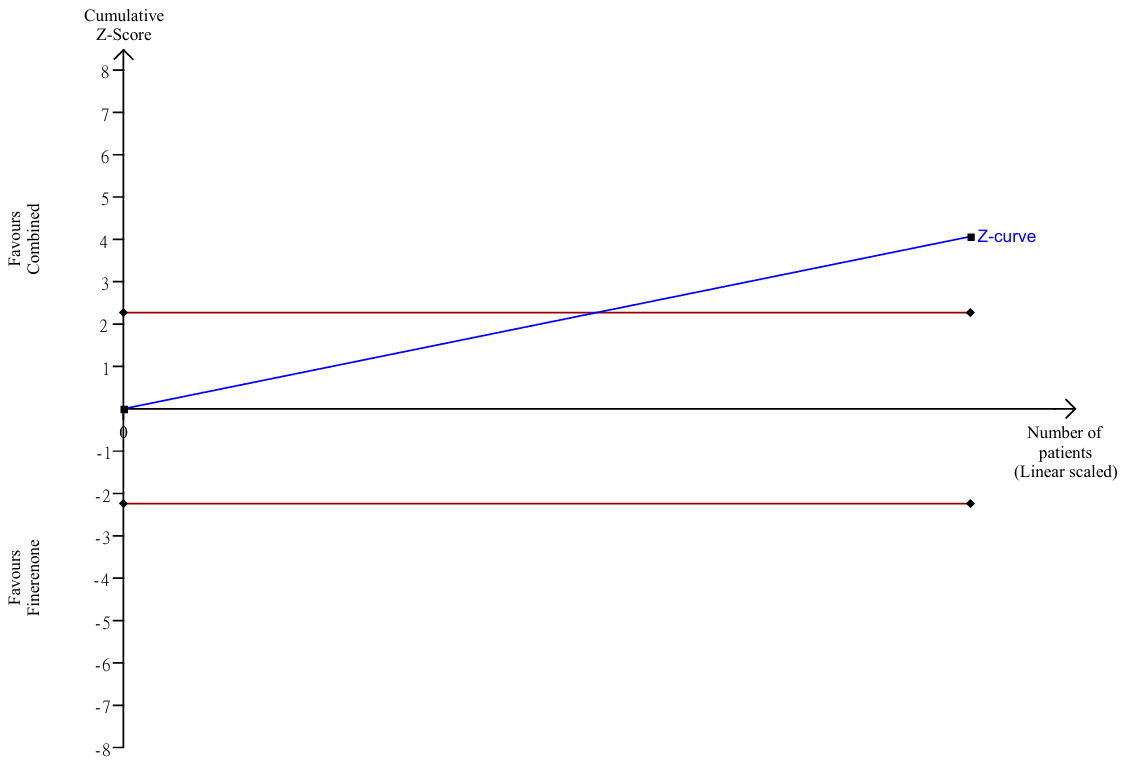
**

**(b)**

**
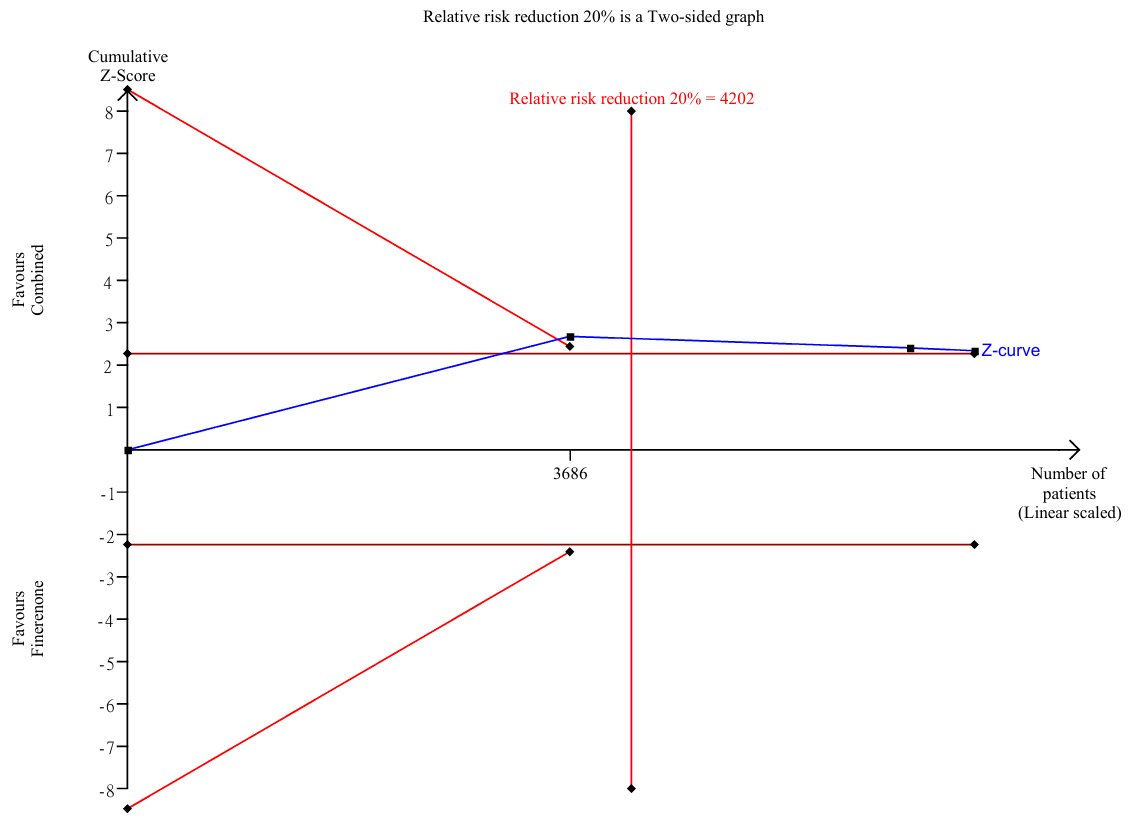
**

**(c)**

**
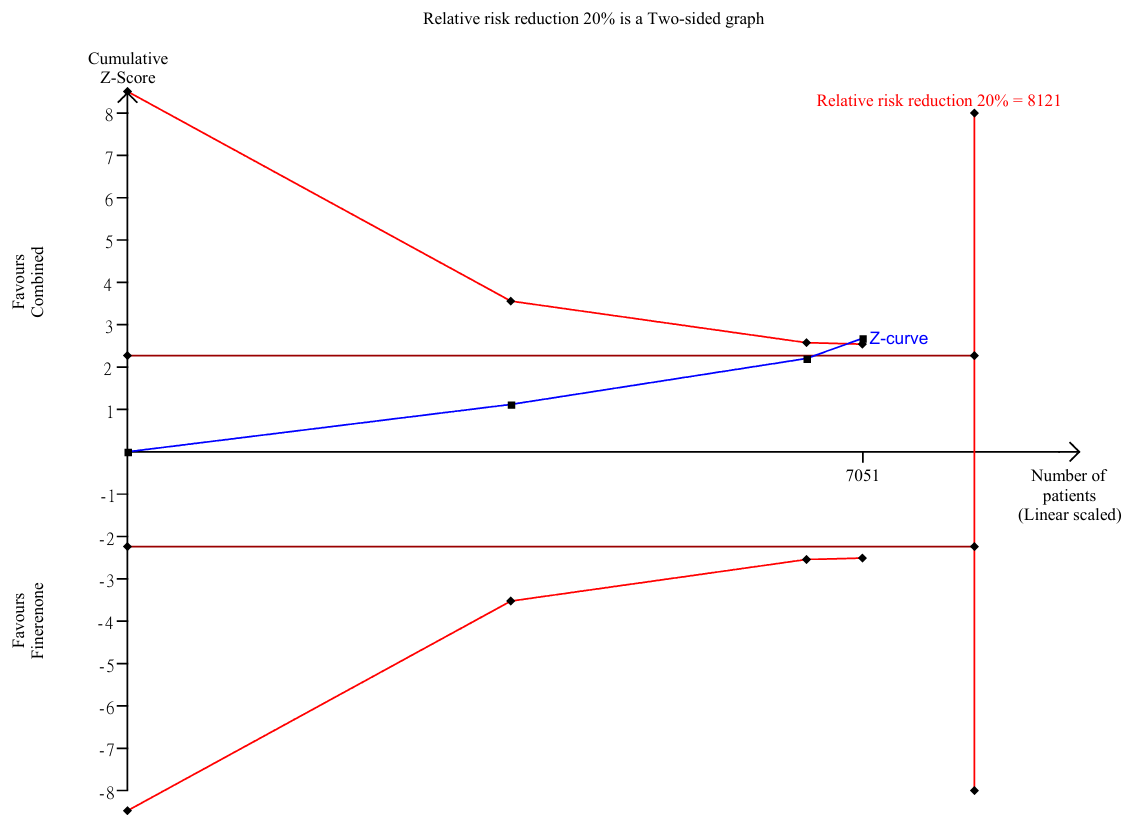
**

**Abbreviations:** MACE, major adverse cardiovascular event; MAKE, major adverse kidney event

**Figure S8 Trial sequential analysis showing a potential risk of bias on the combined therapy versus SGLT2i monotherapy on (a)all-cause mortality, (b) MACE, (c) MAKE**

**(a)**

**
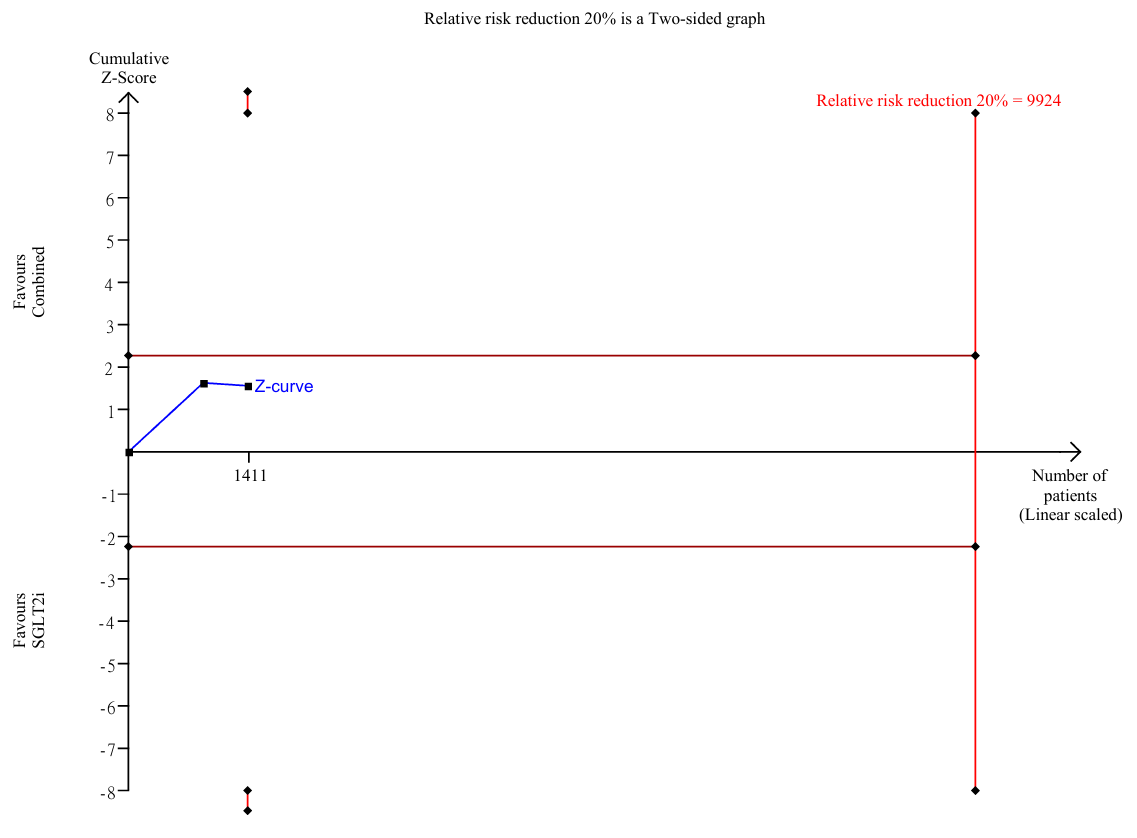
**

**(b)**

**
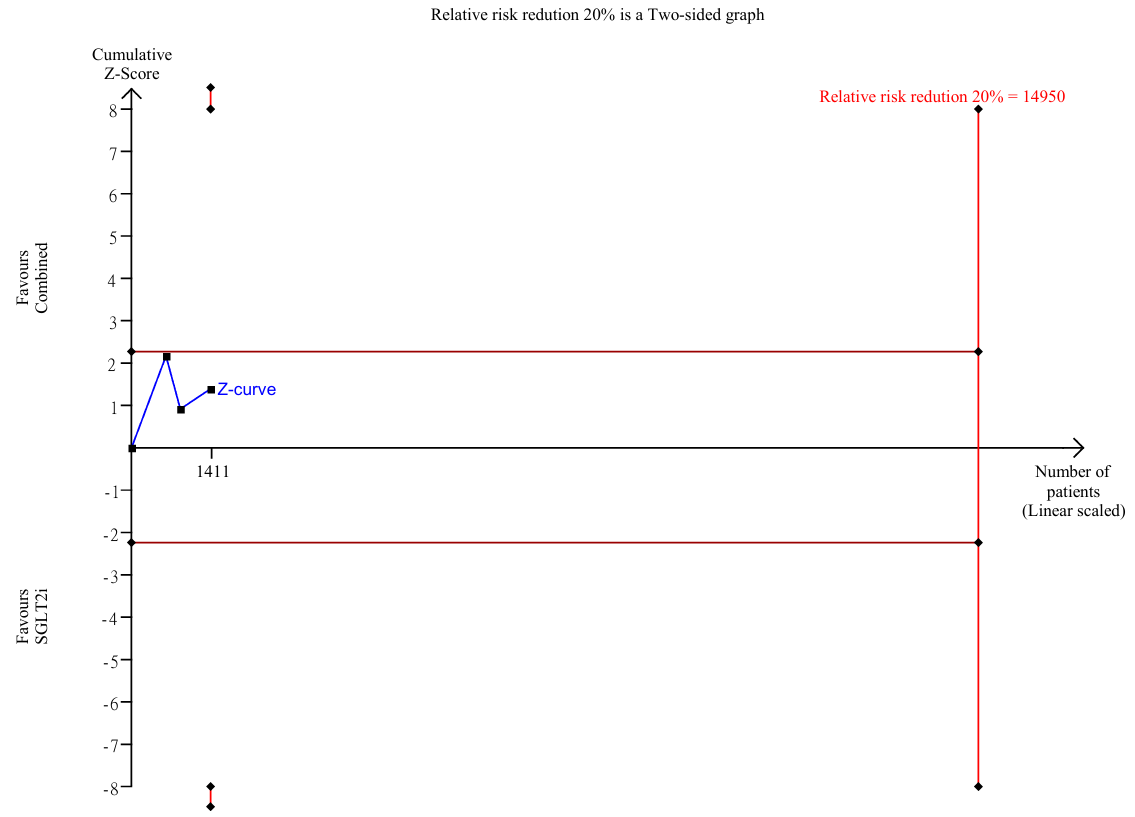
**

**(c)**

**
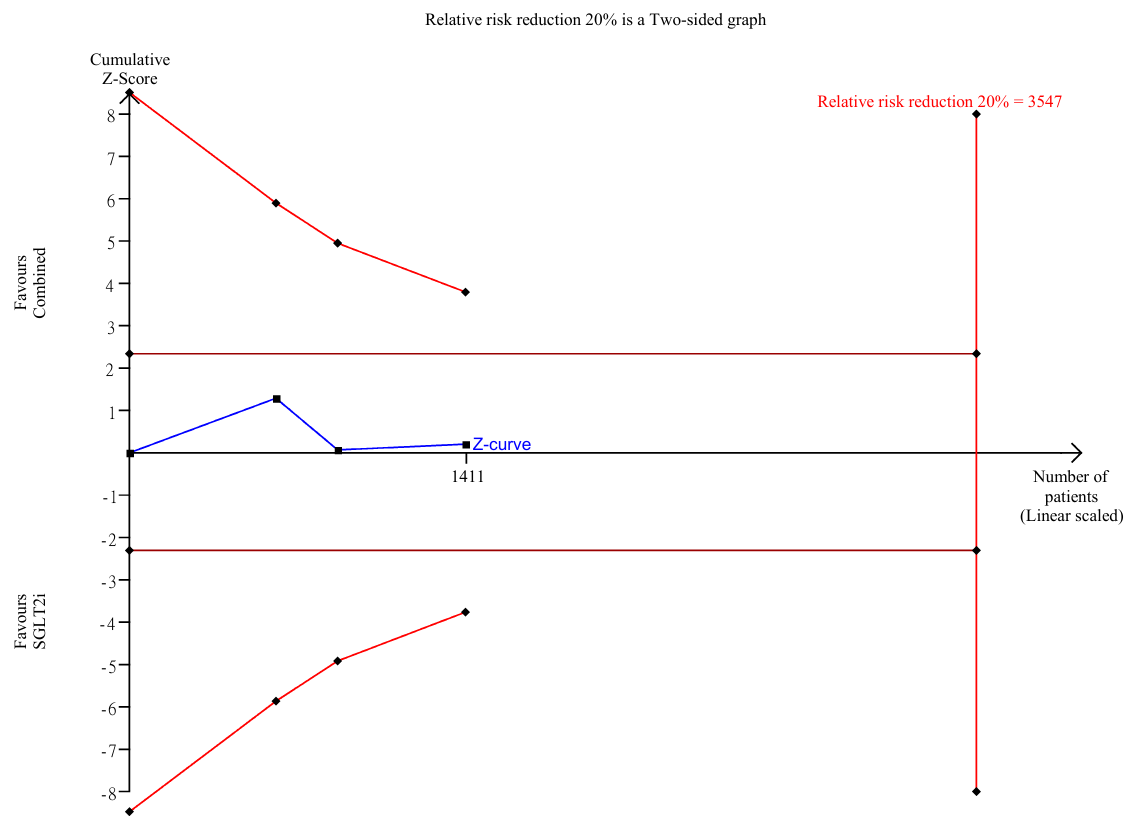
**

**Abbreviations:** MACE, major adverse cardiovascular event; MAKE, major adverse kidney event; SGLT2i, sodium-glucose co-transporter 2 inhibitor
